# Olefin-Surface Interactions: A Key Activity Parameter in Silica-Supported Olefin Metathesis Catalysts

**DOI:** 10.1021/jacsau.2c00052

**Published:** 2022-03-09

**Authors:** Zachariah J. Berkson, Moritz Bernhardt, Simon L. Schlapansky, Mathis J. Benedikter, Michael R. Buchmeiser, Gregory A. Price, Glenn J. Sunley, Christophe Copéret

**Affiliations:** † Department of Chemistry and Applied Bioscience, ETH Zürich, Vladimir-Prelog-Weg 2, Zürich 8093, Switzerland; ‡ Institute of Polymer Chemistry, Universität Stuttgart, Pfaffenwaldring 55, Stuttgart 70569, Germany; § Applied Sciences, BP Innovation & Engineering, BP plc, Saltend, Hull HU12 8DS, U.K.

**Keywords:** heterogeneous catalysis, molybdenum, olefin metathesis, spectroscopy, solid-state NMR, surface organometallic chemistry, molecular dynamics

## Abstract

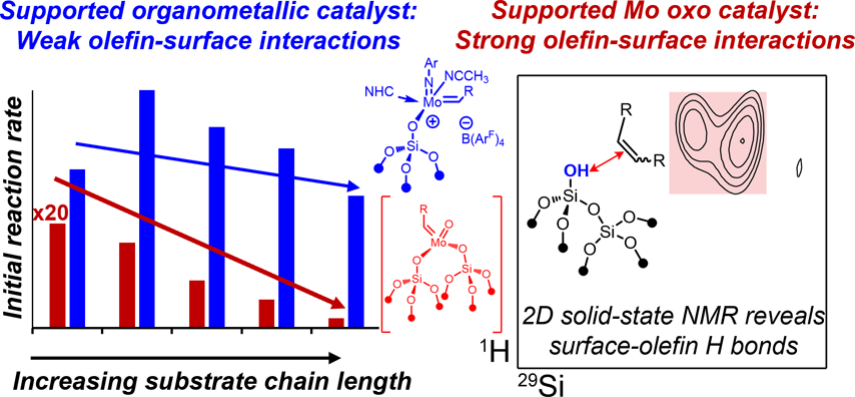

Molecularly defined and classical heterogeneous Mo-based metathesis catalysts are shown to display distinct and unexpected reactivity patterns for the metathesis of long-chain α-olefins at low temperatures (<100 °C). Catalysts based on supported Mo oxo species, whether prepared via wet impregnation or surface organometallic chemistry (SOMC), exhibit strong activity dependencies on the α-olefin chain length, with slower reaction rates for longer substrate chain lengths. In contrast, molecular and supported Mo alkylidenes are highly active and do not display such dramatic dependence on the chain length. State-of-the-art two-dimensional (2D) solid-state nuclear magnetic resonance (NMR) spectroscopy analyses of postmetathesis catalysts, complemented by Fourier transform infrared (FT-IR) spectroscopy and molecular dynamics calculations, evidence that the activity decrease observed for supported Mo oxo catalysts relates to the strong adsorption of internal olefin metathesis products because of interactions with surface Si–OH groups. Overall, this study shows that in addition to the nature and the number of active sites, the metathesis rates and the overall catalytic performance depend on product desorption, even in the liquid phase with nonpolar substrates. This study further highlights the role of the support and active site composition and dynamics on activity as well as the need for considering adsorption in catalyst design.

## Introduction

**Figure 1:**
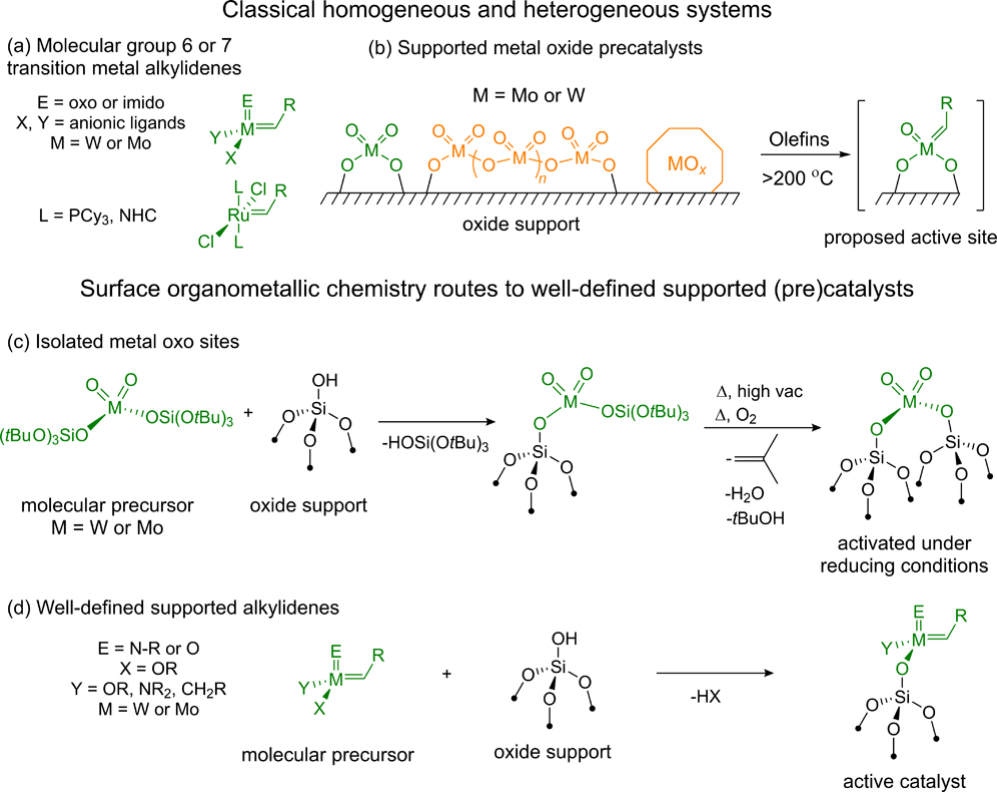
State of the art in olefin metathesis: (a) Molecular group 6 or 7 metal alkylidene olefin metathesis catalysts. (b) Surface species proposed on supported Mo or W oxide precatalysts and the proposed structure of the active site for olefin metathesis. SOMC routes for the preparation of (c) monodispersed metal oxo precatalyst sites or (d) well-defined supported metal alkylidenes.

Olefin metathesis is a key technology for the formation of C=C bonds by the rearrangement of alkylidene fragments among olefins.^1^ Decades of research have yielded highly active and selective olefin metathesis catalysts based on molecular Mo-, W-, and Ru-alkylidenes that are highly active at low temperatures (e.g., room temperature to 100 °C) and tolerant to functional groups in many instances, enabling broad applications in organic and polymer syntheses (Figure 1a).^2−6^ By comparison, heterogeneous olefin metathesis catalysts, mostly based on supported Mo or W oxides, are industrially used for the upgrading of light olefins^7^ but require high-temperature activation and/or reaction conditions (>150 °C and even 400 °C for W-based catalysts).^8^ They are composed of ill-defined surface structures with low (<5%) quantities of active sites and are proposed to require complex initiation processes at high temperatures, involving, for instance, surface OH groups.^9−11^ These shortcomings have limited their broader adoption.

Although the nature of the active sites has remained elusive, it is now accepted that the active sites correspond to high-valent alkylidenes as for their molecular analogues, generated in situ from isolated, high-valent metal oxo species in the presence of olefins (Figure 1b).^8^ Recent work leveraging the principles of surface organometallic chemistry (SOMC)^12,13^ has enabled the generation of atomically dispersed and isolated W(VI) and Mo(VI) oxo sites as models for oxide catalysts prepared through traditional synthetic approaches (Figure 1c).^14,15^ Upon activation in situ with an organosilicon reducing agent, these species display low-temperature activity (<100 °C), originating from the formation of M(IV) species and their in situ conversion to M(VI) oxo alkylidenes upon reaction with the olefin substrate.^14,15^ However, such monodispersed metal oxo catalysts still exhibit significantly lower activity (by several orders of magnitude) than well-defined silica-supported alkylidenes prepared via SOMC (Figure 1d). Given the similar electronic characteristics of surface siloxides and some of the corresponding molecular ligands,^16^ the different reaction patterns are puzzling and suggest that other factors must be at play in addition to the smaller number of active sites in supported metal oxide-based catalysts (5–10% vs ca. 100% for well-defined alkylidenes).^8^ In fact, even well-defined supported alkylidenes can exhibit low catalytic performances in a few instances for metathesis of olefins in the liquid phase. For example, low activity has been observed in ring-closing metathesis reactions^17^ as well as slow initiation for supported cationic W oxo alkylidenes when using very low catalyst loadings.^18^ In both cases, restricted dynamics of surface species have been proposed to explain these reactivity patterns.

In order to expand the application of supported catalysts and to better understand the influences of dynamics on activity, we have investigated the low-temperature (<100 °C) metathesis activities of a series of supported and molecular Mo-based olefin metathesis catalysts toward long-chain linear α-olefins (C_8_–C_20_) of importance to the Shell higher-olefin process (SHOP) and related processes.^19−22^ We observe a surprisingly vast difference in activity patterns among molecular and supported catalysts, including silica-supported and molecular Mo alkylidenes as well as reduced silica-supported Mo oxo species, which reveals the importance of olefin-surface interactions on reactivity. Specifically, the supported Mo oxo systems exhibit strong dependencies of activity as a function of the olefin chain length, in contrast to the well-defined supported or molecular Mo alkylidenes. Solid-state two-dimensional (2D) heteronuclear ^13^C–^1^H and ^29^Si–^1^H NMR correlation analyses of postreaction catalysts, with sensitivity enhanced by state-of-the-art fast magic-angle spinning (MAS) and ^1^H detection^23,24^ or dynamic nuclear polarization (DNP)^25,26^ techniques, complemented by Fourier transform infrared (FT-IR) spectroscopy and molecular dynamics calculations, uncover the strong adsorption of long-chain olefin metathesis hydrocarbon products onto the silica support near surface −OH sites. These interactions result in decreased reaction rates with increasing chain lengths in the case of supported Mo oxides as a result of the stronger adsorption of the internal di-substituted olefin product.

## Results and Discussion

### Activity Trends of Mo Oxo-Based (Pre)Catalysts for Metathesis of Linear α-Olefins

**Figure 2:**
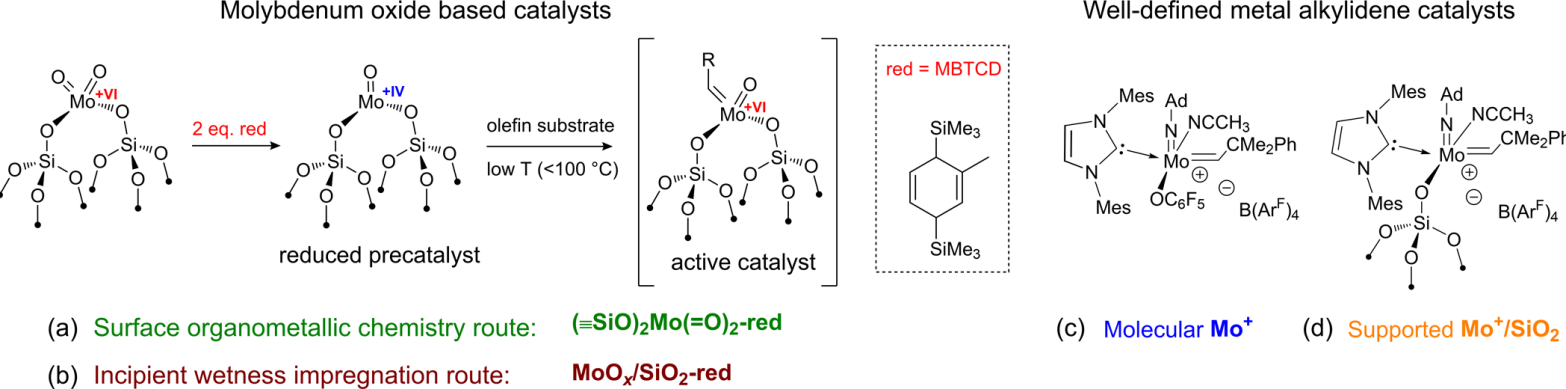
Molybdenum oxide-based catalysts prepared via (a) an SOMC-based approach, **(≡SiO)_2_Mo(=O)_2_-red**, or (b) a conventional incipient wetness impregnation approach, **MoO_*x*_/SiO_2_-red**. Well-defined cationic Mo imido alkylidene NHC catalysts in (c) molecular (**Mo^+^**) and (d) supported (**Mo^+^/SiO_2_**) forms.

We first evaluated the trends in catalyst activity for the metathesis of linear α-olefins for silica-supported Mo oxide-based catalysts. We focused on a broad range of linear α-olefins (C_8_–C_20_) that can be obtained by olefin oligomerization^19,27,28^ or Fischer-Tropsch^21,22,29^ processes. Primarily, molybdenum-based catalysts were tested as they are known to be more efficient for terminal olefin metathesis than their W analogues^30,31^ (*vide infra*) because of better tolerance for ethylene.^32^ Monodispersed Mo dioxo species (1.56 wt % Mo) were generated via an SOMC approach, as previously reported,^15^ and activated at room temperature by prereduction with 2 equiv on a per Mo basis of the molecular organosilicon reductant 1-methyl-3,6-bis(trimethylsilyl)-1,4-cyclohexadiene (MBTCD),^14,33^ yielding a catalyst denoted as **(≡SiO)_2_Mo(=O)_2_-red** (Figure 2a). For comparison, a classical silica-supported Mo oxide catalyst was prepared using an incipient wetness impregnation approach, followed by calcination under synthetic air.^34^ This oxidized precatalyst, which contains 3.65 wt % Mo, was also activated for low-temperature metathesis by 2 equiv of the same organosilicon reductant (MBTCD) and is denoted as **MoO_*x*_/SiO_2_-red** (Figure 2b). Both **(≡SiO)_2_Mo(=O)_2_** and **MoO_*x*_/SiO_2_** contain residual isolated surface Si–OH species, as evidenced by their FT-IR spectra in Figures S1 and S2. Based on previous X-ray absorption spectroscopy (XAS) analyses,^15,35^ both SOMC and incipient wetness impregnation approaches yield predominantly isolated Mo dioxo surface species, although the activity of the resulting precatalysts is modestly different (Table S1), suggesting differences in the quantities of active sites generated.

The reactivities of the catalysts based on metal oxo precatalysts, **(≡SiO)_2_Mo(=O)_2_-red** and **MoO_*x*_/SiO_2_-red**, were assessed at both 70 and 30 °C. All reactions were conducted in batch mode under a N_2_ atmosphere. 1,2-Dichlorobenzene was chosen as the solvent because of its low vapor pressure. Liquid-phase olefin metathesis reaction rates for the metal oxo-based materials were found to generally depend on the precise reaction conditions, particularly the purity of the olefin stock solutions. The highest reaction rates were observed when the olefin substrates were freshly purified immediately before the catalytic reaction tests, according to a rigorous purification protocol (see the Experimental Section for details).^30^

Both **(≡SiO)_2_Mo(=O)_2_-red** and **MoO_*x*_/SiO_2_-red** are competent for the metathesis of linear α-olefins in the liquid phase at low temperatures. For 1-nonene as a prototypical substrate, **(≡SiO)_2_Mo(=O)_2_-red** exhibits maximum product formation rates (measured after ca. 10 min of reaction time with no observed induction period) of 2.6 and 2.0 (mmol product [mmol Mo]^−1^ [min]^−1^) at 70 and 30 °C, respectively (Figures S4 and S5, Table S1). The W-based analogue **(≡SiO)_2_W(=O)_2_-red**(14) was also tested at 70 °C for comparison but was found to exhibit lower activity for 1-nonene metathesis with a maximum rate of 0.9 (mmol product [mmol W]^−1^ [min]^−1^) (Figure S6, Table S1). The lower activity of the W-based catalyst compared to the Mo analogue is consistent with recent studies on well-defined silica-supported alkylidenes,^30,31^ which found that Mo-based catalysts are typically much more active for the metathesis of terminal olefins because of the lower stability of off-cycle square-planar (SP) metallacycle intermediates generated in the presence of ethylene.^32^ By comparison, **MoO_*x*_/SiO_2_-red** exhibits initial 1-nonene product formation rates of 3.9 and 0.71 (mmol product [mmol Mo]^−1^ [min]^−1^) at 70 and 30 °C, respectively (Figures S7 and S8, Table S1). For both **(≡SiO)_2_Mo(=O)_2_-red** and **MoO_*x*_/SiO_2_-red**, significant conversion (6–11%) is observed to internal olefin isomers of the desired 1-nonene metathesis product at 70 °C (Table S1), suggesting the formation of Mo hydrides that promote the isomerization of the long-chain internal olefins.^36^ Improved product selectivities were observed at 30 °C (>98%) compared to 70 °C (Table S1). Additionally, substantial solvent evaporation was observed at 70 °C after long reaction times when open reaction vials were used to allow for the release of ethylene. Accordingly, reactions were also run at 70 °C in closed reaction vials, yielding higher initial reaction rates but lower overall conversions (Table S2). The product *E* and *Z* selectivities of the two catalysts based on silica-supported Mo oxo species, **(≡SiO)_2_Mo(=O)_2_-red** and **MoO_*x*_@SiO_2_-red,** were very similar at low conversions, ca. 70 and 30%, respectively (*E*/*Z* ratio ∼2.1 at 70 °C and ∼2.3 at 30 °C, Figure S9 and S10, Table S1). Because stereoselectivity in olefin metathesis at low conversions depends directly on the structure of the active site,^37^ these similar values corroborate that both catalysts have similar active site structures.

**Figure 3:**
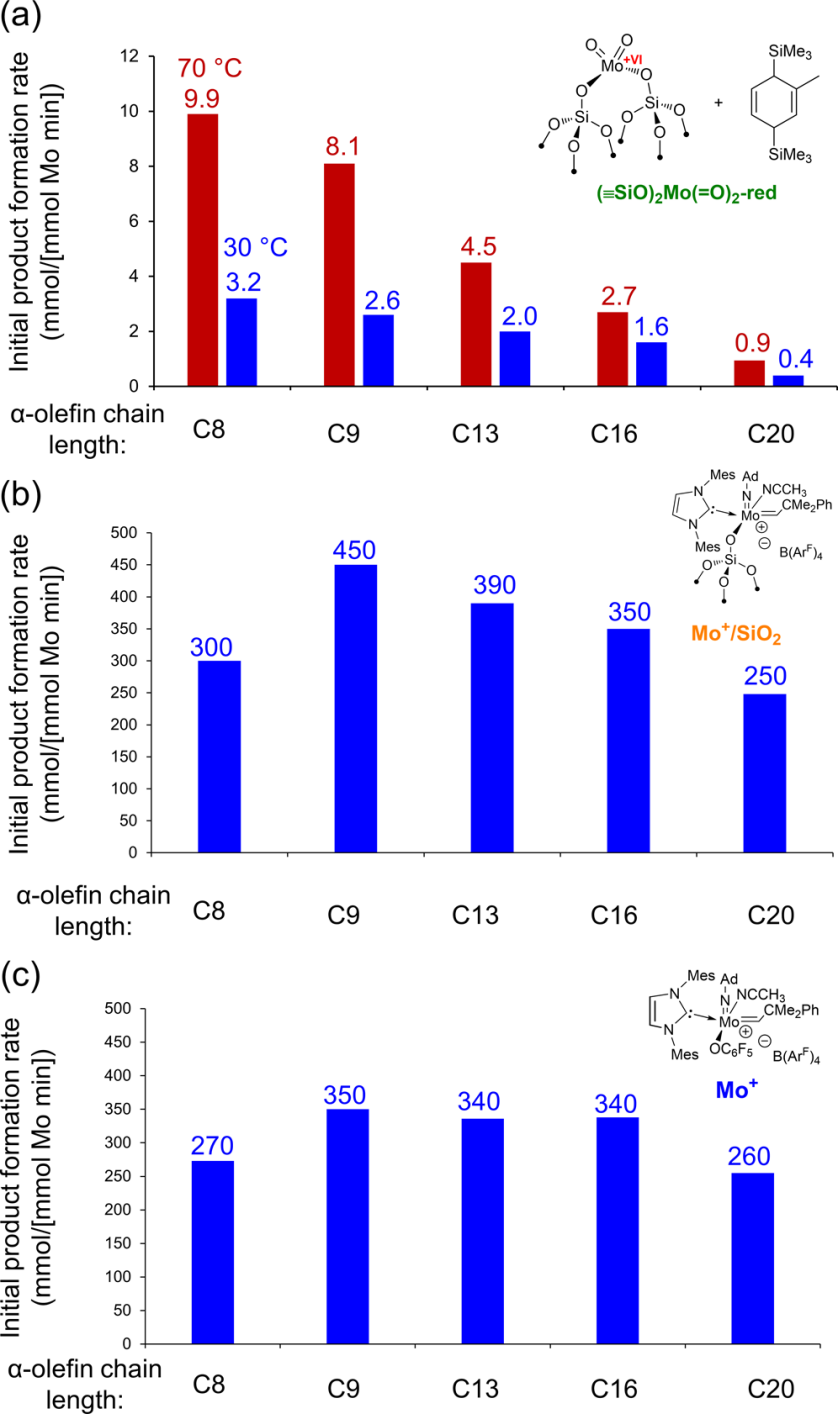
Initial product formation rates for the metathesis of linear alpha olefins catalyzed by (a) **(≡SiO)_2_Mo(=O)_2_-red** at 70 °C (red) and 30 °C (blue) or well-defined Mo imido alkylidene NHC catalysts (b) **Mo^+^/SiO_2_** or (c) molecular **Mo^+^**. All reactions were carried out under an N_2_ atmosphere, in closed (70 °C) or open (30 °C) batch reactors containing 2.5 mL of substrate stock solution in 1,2-dichlorobenzene. 1 M substrate stock solutions were used for 1-octene, 1-nonene, 1-tridecene, and 1-hexadecene, with substrate: Mo ratios of ca. 1000:1 for **(≡SiO)_2_Mo(=O)_2_-red** and ca. 5000:1 for **Mo^+^** and **Mo^+^/SiO_2_**. For 1-eicosene, a 0.5 M substrate stock solution was used with substrate: Mo ratios of ca. 500:1 for **(≡SiO)_2_Mo(=O)_2_-red** and ca. 2500:1 for **Mo^+^** and **Mo^+^/SiO_2_**.

To assess the influence of the olefin chain length on activity, **(≡SiO)_2_Mo(=O)_2_-red** was tested as a catalyst for the metathesis of the linear α-olefins 1-octene, 1-nonene, 1-tridecene, 1-hexadecene, and 1-eicosene. Maximum product formation rates of 9.9, 7.8, 4.5, 2.7, and 0.9 (mmol product [mmol Mo]^−1^ [min]^−1^) at 70 °C (in closed reaction vials) and 3.2, 2.6, 2.0, 1.6, and 0.4 (mmol product [mmol Mo]^−1^ [min]^−1^) at 30 °C (in open reaction vials) were observed after 10 min reaction time. Lower substrate concentrations (0.5 M) were used for the 1-eicosene reaction tests to mitigate the poor solubility of the very long-chain metathesis product. The initial product formation rates are compared in Figure 3a, the kinetic profiles are shown in Figures S11–S20, and catalytic reaction data are summarized in Tables S2 and S3. **MoO_*x*_@SiO_2_-red** was also tested for 1-nonene, 1-tridecene, and 1-hexadecene metathesis at 30 °C, and it showed trends similar to **(≡SiO)_2_Mo(=O)_2_-red**, although with somewhat lower activity on a per Mo basis and, in some cases, an induction period (Figures S21–S23, Table S4). As shown in Figure 3a, reaction rates for **(≡SiO)_2_Mo(=O)_2_-red** decrease monotonically as a function of the olefin chain length for the entire substrate series studied here at both 70 and 30 °C.

While **(≡SiO)_2_Mo(=O)_2_-red** exhibits overall promising activity and selectivity in the metathesis of linear α-olefins at a low temperature (<100 °C), we sought to understand the origin of its reduced metathesis activity for long-chain, terminal α-olefins. We hypothesized three possible explanations: Hypothesis (A): the intermediates on the catalytic cycle are energetically disfavored for longer chain olefins, for example, because of steric factors; Hypothesis (B): initiation of the Mo(IV) oxo species to form Mo(VI) oxo alkylidenes is chain-length-dependent; or Hypothesis (C): there is chain-length dependence for the interactions of olefinic products and the catalyst surface that influences reactivity. In the case of Hypothesis (B), we would expect similar maximum product formation rates for all olefins, with different induction periods because of different rates of catalyst initiation. As no induction periods are observed for this catalyst, Hypothesis (B) seems unlikely. To investigate Hypothesis (A), we measured the reactivity trends of well-defined metal alkylidene-based catalysts in molecular and supported forms.

### Activity of Well-Defined Molecular and Supported Mo Alkylidene Olefin Metathesis Catalysts

To assess the influence of the active site structure and surface composition on linear α-olefin metathesis activity, we tested for comparison a well-defined and highly active cationic Mo imido alkylidene *N*-heterocyclic carbene (NHC) catalyst in both molecular (**Mo^+^**)^38^ and silica-supported (**Mo^+^/SiO_2_**)^39^ forms (Figure 2c,d). By comparison to the catalysts based on supported Mo oxides, those based on well-defined Mo alkylidenes (**Mo^+^** and **Mo^+^/SiO_2_**) exhibited orders of magnitude higher activity at 30 °C with no induction period, which is consistent with the presence of the initiating alkylidene ligand and the optimized ligand sets of these catalysts. While the increased reaction rates can be due to the specific nature and the number of active sites, the different trends in reactivity as a function of olefin chain length are noteworthy. Specifically, the initial product formation rates for **Mo^+^/SiO_2_** (measured after 3 min reaction time) vary only slightly from 300, 450, 380, 340, and 250 (mmol product [mmol Mo]^−1^ [min]^−1^) for 1-octene, 1-nonene, 1-tridecene, 1-hexadecene, and 1-eicosene, respectively (Figure 3b). By comparison, the molecular catalyst **Mo^+^** exhibited similar maximum product formation rates (measured after 3 min reaction time) of 270, 350, 340, 340, and 260 (mmol product [mmol Mo]^−1^ [min]^−1^) for 1-octene, 1-nonene, 1-tridecene, 1-hexadecene, and 1-eicosene, respectively (Figure 3c). Additional details of the catalytic tests are provided in Figures S24–S33 and Tables S5 and S6. Based on repeated measurements, the uncertainty of the product formation rates was estimated to be ±30 (mmol product [mmol Mo]^−1^ [min]^−1^) (see Figure S33 for details). In general, the well-defined cationic alkylidene catalysts were highly efficient in both molecular and supported forms: for each of the linear α-olefin substrates tested, **Mo^+^** and **Mo^+^/SiO_2_** reached >40% conversion within the first 3 min of the reaction period. The supported catalyst **Mo^+^/SiO_2_** exhibited equivalent or higher activity for each substrate compared to **Mo^+^**, which is consistent with previous comparisons of well-defined supported and molecular alkylidenes.^13,39^ In contrast to **(≡SiO)_2_Mo(=O)_2_-red** and **MoO_*x*_@SiO_2_-red**, the molecular catalyst **Mo^+^** showed little dependence of activity on the substrate chain length, with a slight optimum for olefins in the C9–C16 range. Thus, there seems to be no intrinsic limitation for the metathesis of long-chain olefins related to the catalytic intermediates themselves, enabling us to discard Hypothesis (A) posed above. **Mo^+^/SiO_2_** exhibited an optimum activity for 1-nonene, as well as a modest decrease in activity as a function of the substrate chain length from 1-nonene to 1-eicosene, although not as pronounced as that observed for **(≡SiO)_2_Mo(=O)_2_-red** at 30 °C (Figure S34). Overall, the reactivity trend of **Mo^+^/SiO_2_** appears closer to that of molecular **Mo^+^** than the supported metal oxide catalysts. Based on these observations, we investigated Hypothesis (C) that the reaction trends of the supported Mo oxo and alkylidene-based catalysts arise in large part from differences in surface dynamics and olefin adsorption relating to the distinct surface compositions of the catalysts, which could also account for the decreasing activity with olefin chain length of the Mo oxide systems.

### Metathesis Products Adsorbed on Postreaction Metathesis Catalysts

**Figure 4:**
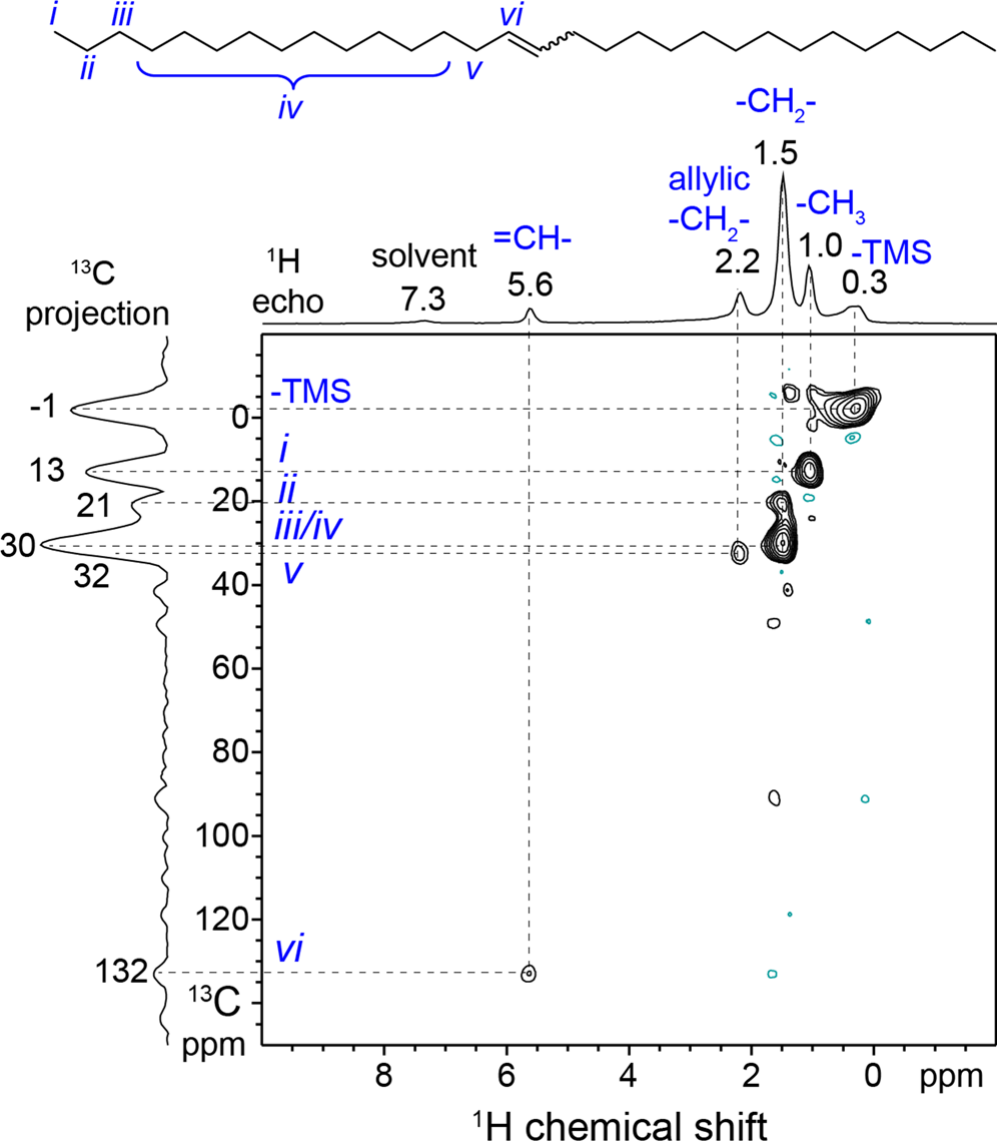
Solid-state 2D ^1^H{^13^C} *D*-HMQC NMR correlation spectrum of **(≡SiO)_2_Mo(=O)_2_-red** after 24 h reaction with 1-hexadecene in 1,2-dichlorobenzene at 30 °C (ca. 80% conversion), 3× washing with C_6_H_6_, and drying under high vacuum. The spectrum was acquired at 16.4 T, 50 kHz MAS, 280 K, and with 60 rotor periods (1.2 ms) for ^13^C–^1^H recoupling. A 1D ^1^H echo MAS NMR spectrum acquired under the same conditions is shown along the horizontal axis for comparison. All correlated signals are assigned to surface trimethylsilyl (-TMS) or to the internal olefin product of 1-hexadecene self-metathesis as indicated by the Roman numeral labels on the molecular structure above.

To assess the adsorption and interactions of olefins on these catalysts, FT-IR and solid-state NMR spectroscopies were used to probe the structure, dynamics, and interactions of the organics on the supported catalysts after reaction. FT-IR and one-dimensional (1D) ^1^H solid-state nuclear magnetic resonance (NMR) analyses of **(≡SiO)_2_Mo(=O)_2_-red** after reaction show the presence of organic species (Figures S35–S37), which increase in relative quantity as a function of reaction time. This is corroborated by elemental analysis, which further indicates that the surface coverage of organic approaches monolayer coverage as reaction time increases (Table S7). Solid-state 2D heteronuclear correlation NMR spectra were therefore acquired to establish the types of organic species and their surface interactions by leveraging NMR sensitivity enhancements provided by either fast-MAS and ^1^H detection^23,24^ or by DNP.^25,26^ For example, Figure 4 shows the solid-state 2D ^1^H{^13^C} dipolar heteronuclear multiple quantum correlation (*D*-HMQC) spectrum of **(≡SiO)_2_Mo(=O)_2_-red** after 24 h reaction at 30 °C with 1-hexadecene in 1,2-dichlorobenzene (ca. 80% conversion). Prior to the solid-state NMR analysis, the catalyst was washed with benzene to remove weakly bound surface species and dried under high vacuum (see the Experimental Section for details). Fast-MAS (50 kHz) and indirect detection provide high ^13^C NMR sensitivity and resolution, enabling the detection of the 2D spectrum of the surface-bound organic species at natural abundance (1.1%) ^13^C. The 2D ^1^H{^13^C} spectrum shows well-resolved correlated signals that can each be assigned to organic moieties on the catalyst surface. Specifically, the correlated signal at −1 ppm in the ^13^C dimension and 0.3 ppm in the ^1^H dimension is assigned to surface −OSi(CH_3_)_3_ moieties resulting from the reaction of the organosilicon reductant. The ^1^H signals at 1.0, 1.5, and 2.2 ppm are each correlated to ^13^C signals at 13, 21–30, and 32 ppm, respectively, which are assigned to −CH_3_, aliphatic −CH_2_–, and allylic −CH_2_– moieties, respectively, while the ^1^H signal at 5.6 ppm is correlated to a ^13^C at 132 ppm and is assigned to internal olefinic species. The absence of ^13^C or ^1^H signals from other olefinic species and the relatively narrow linewidth of the ^1^H signal at 5.6 ppm indicate that only a single type of internal olefin is present at the catalyst surface, most likely the bulky C30 product of 1-hexadecene metathesis, 15-triacontene. Differences between the *E-* and *Z*-stereoisomers cannot be resolved in this case as they are expected to be separated by <0.1 ppm in ^1^H NMR and <0.5 ppm in ^13^C NMR. This internal olefin is strongly adsorbed on to the catalyst surface, as further corroborated by ^1^H *T*_2_ spin–spin relaxation time analyses (Table S8), which are sensitive to the dynamics of surface species.^40^ The *T*_2_ relaxation times of the adsorbed olefins are found to be quite short (<2 ms), consistent with strong adsorption and hindered dynamics of the surface-bound organics. The 1D and 2D ^1^H{^13^C} MAS NMR spectra and analyses thus establish that the predominant surface-bound organic component on postreaction **(≡SiO)_2_Mo(=O)_2_-red** is the bulky olefin metathesis product, which adsorbs strongly under mild reaction conditions, thereby limiting the catalyst efficiency.

This adsorption of the olefin metathesis products appears to be competitive with the 1,2-dichlorobenzene solvent. This is evidenced by the comparison of the ^1^H MAS NMR spectra of **(≡SiO)_2_Mo(=O)_2_-red** after different reaction times with 1-hexadecene or 1-nonene, as shown in Figures S36 and S37. The spectra exhibit a ^1^H signal at 7.3 ppm, which is assigned to residual adsorbed 1,2-dichlorobenzene solvent. However, for the catalyst after 1-nonene metathesis, this signal was greatly increased in intensity relative to the ^1^H signals from adsorbed olefinic species, while for the catalyst after 1-hexadecene metathesis, the solvent signal was greatly diminished. After increasing 1-hexadecene metathesis reaction times, the signal from dichlorobenzene decreases in intensity relative to the signals from adsorbed olefins (Figure S36), which is consistent with the increased relative coverage of olefin species.

The nature of the olefin-surface interaction was further elucidated by the analysis of 2D ^29^Si{^1^H} and ^13^C{^1^H} heteronuclear correlation (HETCOR) spectra leveraging DNP–NMR techniques at low temperatures. DNP–NMR provides greatly enhanced NMR signal sensitivity from surfaces and enables the acquisition of 2D NMR correlation spectra that probe organic–inorganic interactions of adsorbed and surface species.^41,42^ Although DNP–NMR techniques have been widely used for the analysis of diverse organic–inorganic hybrid materials, including colloidal nanoparticles^43^ and catalysts,^44,45^ there are surprisingly few examples of its application to characterize molecular adsorption phenomena at surfaces,^46^ despite the critical importance of such phenomena for catalysis.

The DNP-enhanced 1D ^13^C{^1^H} CP-MAS spectrum of **(≡SiO)_2_Mo(=O)_2_-red** after 4 h reaction with 1-hexadecene (Figure S38) shows similar ^13^C signals to those observed in Figure 3, although slightly broader because of slower molecular dynamics under the low-temperature conditions.^40^ In addition to the ^13^C signal at 128 ppm of internal olefinic and/or aromatic species, a ^13^C signal is detected at 116 ppm from terminal olefinic moieties, evidencing the coexistence of a distribution of olefin species adsorbed on the catalyst surface at intermediate reaction times (i.e., low conversions), consistent with the room-temperature 1D ^1^H MAS NMR analyses (Figure S36). This suggests that the metathesis activity of liquid-phase olefins at low temperatures is largely mediated by the desorption of the internal olefin product, with a pool of adsorbed olefin molecules building up on the catalyst surface at longer reaction times.

**Figure 5:**
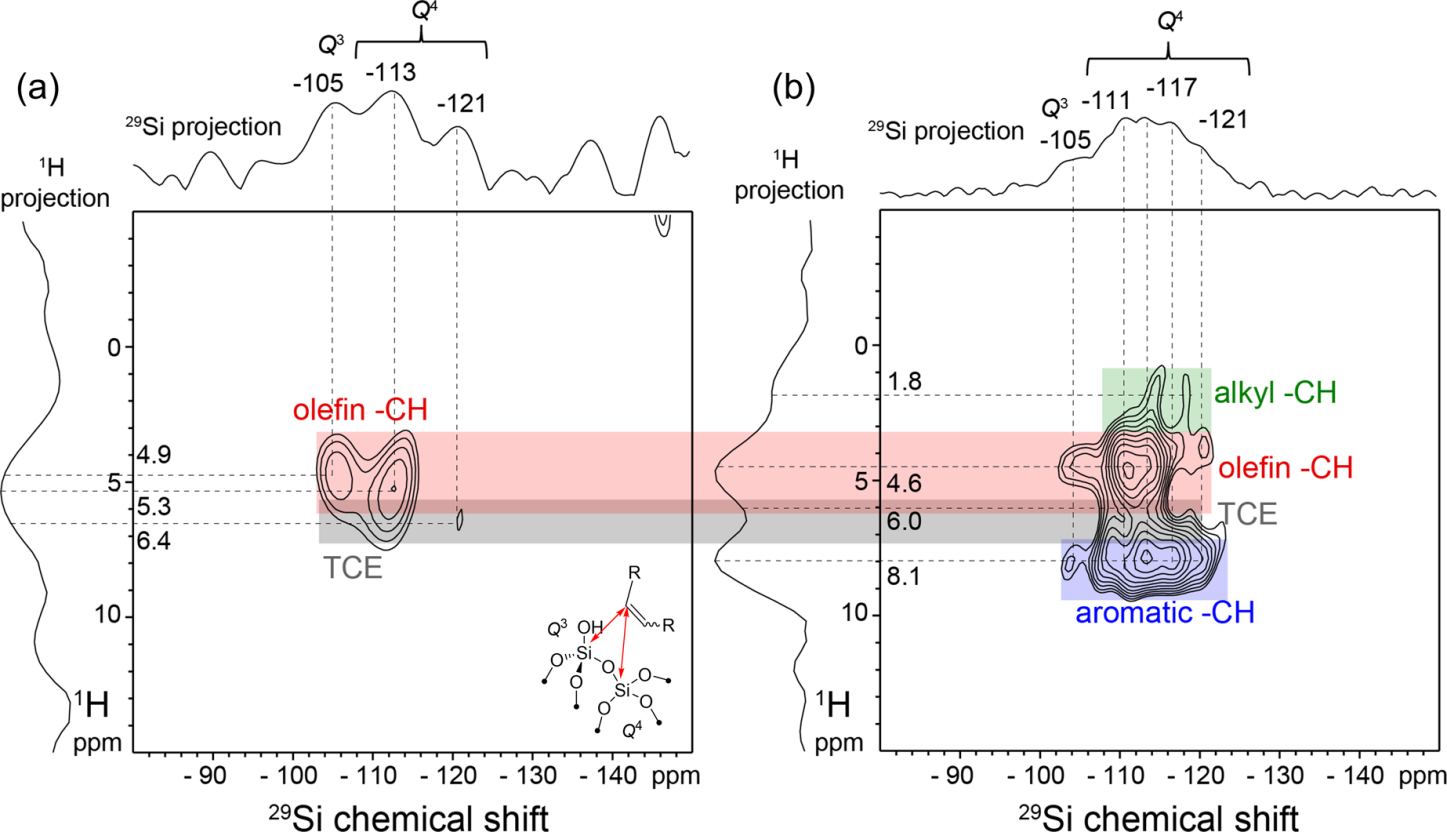
Solid-state 2D ^29^Si{^1^H} DNP-HETCOR spectra of **(≡SiO)_2_Mo(=O)_2_-red** after 4 h reaction with 1-hexadecene in 1,2-dichlorobenzene at 30 °C, 3× washing with C_6_H_6,_ and drying under high vacuum. The 2D spectra were acquired at 14.1 T, 12.5 kHz MAS, 100 K, under continuous microwave irradiation at 395 GHz, in the presence of 16 mM TEKPol biradical in 1,1,2,2-tetrachloroethane (DNP solvent), and with ^29^Si–^1^H contact times of (a) 0.5 ms and (b) 5 ms. Schematic inset in (a) shows the interactions of an olefin moiety with surface Si–OH (*Q*^3^) and fully cross-linked (*Q*^4^) surface silicate species, consistent with the correlated signals in the 2D spectra.

Specifically, surface Si–OH species are directly identified as olefin adsorption sites in **(≡SiO)_2_Mo(=O)_2_-red** by the analysis of 2D ^29^Si{^1^H} DNP-HETCOR spectra shown in Figure 5, which probe ^29^Si–^1^H interactions over subnanometer length scales that vary as a function of ^29^Si–^1^H contact time. The different ^1^H signals are resolved and assigned based on the 2D ^13^C{^1^H} HETCOR spectrum shown in Figure S38 (see the Supporting Information for details). At short ^29^Si–^1^H contact times (0.5 ms, Figure 5a), weak ^29^Si signals are detected at −105, −113, and −121 ppm, which are assigned based on the literature^47,48^ to partially cross-linked *Q*^3^ species and two different types of fully cross-linked surface *Q*^4^ species, respectively. The *Q^n^* notation indicates a silicon atom in a tetrahedral environment bonded to four oxygen atoms, of which *n* are bonded to another silicon atom and 4 – *n* are incompletely cross-linked, for example, H-terminated. Notably, the ^29^Si signals at −105 and −113 ppm are correlated with ^1^H signals at 4.9 and 5.3 ppm from olefinic species, which directly establishes the subnanometer proximities and mutual interactions of surface silanols and olefinic moieties of adsorbed molecules, as depicted schematically in the inset of Figure 5a. The short contact times used make this measurement principally sensitive to interactions over distances of <0.5 nm, indicating that the olefinic moieties of the surface-bound olefins interact preferentially with surface silanol species over subnanometer distances. This is consistent with weak H bonds between the surface silanols and adsorbed olefins, similar to what has been proposed for olefin-methanol H bonds in solution.^49^ Indeed, π–H bonds have recently been observed experimentally for olefins adsorbed on hydroxylated silica surfaces.^50,51^ The participation of surface Si–OH groups in olefin adsorption is confirmed by FT-IR spectroscopy, which shows an increase in the intensity of broad signals from interacting Si–OH groups as a function of reaction time, with a concomitant decrease in the intensity of the signal from isolated Si–OH groups (Figure S35).

The 2D ^29^Si{^1^H} DNP-HETCOR spectra also corroborate the presence of coadsorbed dichlorobenzene molecules and trimethylsiloxy surface moieties. At longer ^1^H–^29^Si contact times (5 ms, Figure 5b), additional correlated signals are detected at 1.8 and 8.1 ppm, which arise, respectively, from alkyl and aromatic ^1^H species, consistent with both the close proximity of the aliphatic chains of the long-chain olefins at the silica surface and the coadsorption of 1,2-dichlorobenzene molecules. Weak ^29^Si signals are also detected at 28 and 22 ppm (Figure S39), which are assigned based on their chemical shift positions to two different types of surface −OSi(CH_3_)_3_ that are byproducts of the decomposition of the organosilicon reductants.

### Dynamics and Adsorption of Olefins on Silica

Overall, the solid-state NMR and FT-IR results and analyses evidence that long-chain internal olefin metathesis products adsorb competitively with solvent molecules at surface Si–OH sites. Such substrate-surface interactions have not previously been the subject of detailed analysis in the field of olefin metathesis, and indeed are unexpected for liquid-phase catalysis with nonpolar substrates in a polar solvent. However, it has been recognized previously that adsorption of olefins importantly influences activity and selectivity for gas-phase catalysis. For instance, strong adsorption of olefins on alumina favors secondary metathesis isomerization reactions in the CH_3_ReO_3_/Al_2_O_3_ system, leading to thermodynamic product selectivities for propene metathesis across a broad range of contact times.^37,52^ The modification of the alumina surface to passivate surface Al-OH moieties yields nonequilibrium *E*/*Z* selectivities.^52^ While alumina and silica-alumina materials are well known to strongly adsorb olefins,^53^ silica is typically considered a more inert support because of the absence of strong Brønsted or Lewis acid sites. However, there is growing recognition of the importance of surface interactions in mediating reactivity, particularly for challenging substrates. For instance, interactions of functionalized olefins containing ester groups and surface silanol groups were recently found to enrich the near-surface concentration of olefins and influence product selectivities for ring-closing metathesis reactions catalyzed by well-defined cationic Mo alkylidenes supported on mesoporous silicas.^54^ The silica-supported Mo oxo system is known to possess strong Brønsted acid sites that could act as adsorption sites,^11,55^ and even on bare hydroxylated silica, the interaction energies of hydrocarbons are known to increase as a function of the chain length and are greater for alkenes than alkanes.^56^ The catalytic reaction tests and spectroscopic analyses discussed above show that substrate-silica interactions are non-negligible for long-chain olefinic hydrocarbons and indeed have significant effects on catalytic reaction properties at low reaction temperatures (<100 °C).

**Figure 6:**
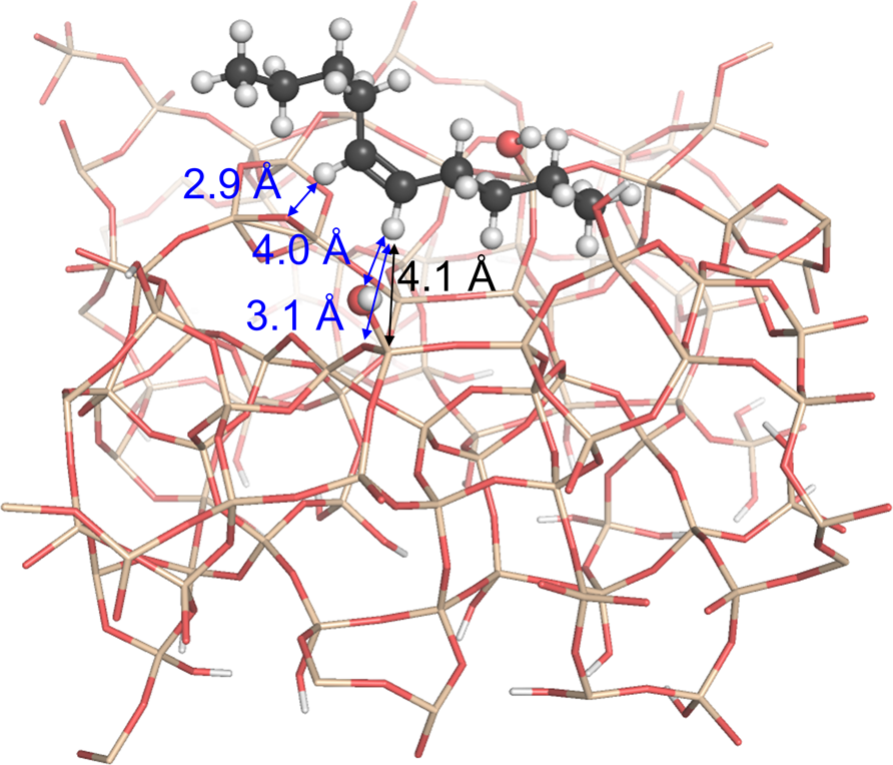
Representative conformation from MD simulations of an internal olefin (*cis*-5-decene) adsorbed on a model surface of dehydroxylated silica. The three shortest CH–O distances are indicated with blue arrows, and the black arrow indicates an olefinic CH to *Q*^3^ Si distance consistent with the 2D solid-state ^29^Si{^1^H} DNP-HETCOR NMR spectrum in Figure 5a.

To assess further the nature of the olefin-surface interactions, we contacted dehydroxylated silica SiO_2–700_ with 1-hexadecene and its metathesis product, 15-triacontene (see the Supporting Information for details). IR and solid-state ^1^H NMR spectra of the washed and dried materials (Figures S40 and S41) show that both olefins adsorb appreciably on the bare silica surface, although the C30 metathesis product to a much greater extent, with concomitant perturbation of the IR signal from isolated Si–OH groups. To gain more insights into these effects, we conducted molecular dynamics (MD) calculations of internal and terminal linear olefins of varied chain lengths on a periodic surface model of dehydroxylated amorphous silica having approximately 1.1 OH/nm^2^.^57^ In each case, the olefins appear to be stabilized on the surface by interactions with surface silanols, with the distances between surface OH and olefinic carbon atoms ranging from 0.22 and 0.55 nm (Table S9), consistent with the solid-state NMR results discussed above. A representative conformation is shown in Figure 6. These distances are within the range expected for olefin-OH hydrogen bonding interactions.^49^ Short distances (0.24 to 0.36 nm) are also observed between allylic and aliphatic H atoms and surface siloxane bridges, suggesting that dispersion interactions with surface siloxanes provide additional stabilization of the adsorbed olefins at the surface, increasing in strength as a function of the chain length. Indeed, the magnitude of the calculated energies of adsorption generally increases as a function of the chain length for both terminal and internal olefins (Table S9), with internal olefins exhibiting slightly stronger adsorption energies compared to terminal olefins of the same molecular weight. These trends are qualitatively consistent with the measurements of olefin adsorption energies and enthalpies on silica by gas chromatography.^56^

Based on the spectroscopic and MD analyses, the stabilization of olefins at the surface of silica is due to hydrogen bonding and van der Waals interactions between the olefins and surface Si–OH and siloxane moieties; the increase in adsorption energy follows the olefin chain length and reflects the increasing contribution of dispersion forces. FT-IR analyses of **(≡SiO)_2_Mo(=O)_2_-red** after reaction, as well as dehydroxylated silica devoid of Mo centers contacted with different olefins, corroborate that isolated Si–OH groups are primarily responsible for the observed olefin-surface interactions (Figures S35 and S40). By comparison, **Mo^+^/SiO_2_** exhibits only broadened and displaced FT-IR signals from SiOH species interacting with organic moieties, which are minimally perturbed on the postreaction catalyst (Figure S42), suggesting that they participate to a lesser extent in olefin adsorption. Indeed, no resolved ^1^H NMR signals from adsorbed olefin are observed in the ^1^H MAS NMR spectrum of **Mo^+^/SiO_2_** postreaction, although because such signals would overlap in part with the ^1^H signals from the organic ligands, we cannot preclude the adsorption of olefins to some degree. Overall, these observations suggest that the adsorption of bulky di-substituted and consequently more electron-rich olefin metathesis products on the catalyst surface, to which we attribute the decreasing activity with the substrate chain length of **(≡SiO)_2_Mo(=O)_2_-red**, depends primarily on the quantities and types of Si–OH groups.

## Conclusions

Silica-supported Mo-based catalysts are active and selective for low-temperature (<100 °C) metathesis of linear α-olefins in the liquid phase, with reaction properties that depend strongly on the characteristics of both the catalyst and the substrate, decreasing sharply as a function of olefin chain length for supported Mo oxo-based catalysts. By comparison, molecularly defined alkylidene catalysts, whether homogeneous or silica-supported, display very high reaction rates (>250 min^–1^) with much less dependence on the olefin chain length. FT-IR and solid-state NMR analyses of catalysts postmetathesis show that the internal olefin metathesis products adsorb on the catalyst support via interactions of olefinic moieties and surface (OH) functionalities; this correlates with the decreased activity of the Mo oxo based catalysts. The observations are further corroborated by MD calculations. Overall, the analyses indicate that the metathesis rate of long-chain linear liquid α-olefins can be limited by the desorption of the bulky internal olefin products from the solid catalyst even in the condensed phase for metathesis catalysts based on supported Mo oxides, prepared either via SOMC or classical wet impregnation approaches. This study also shows the utility of sensitivity-enhanced solid-state NMR as a tool for elucidating surface interactions in heterogeneous systems and understanding the molecular-scale origins of catalytic reaction properties. In addition to offering insights into the origins of the lower activity for supported catalysts based on metal oxides, our study highlights the advantages of molecularly defined supported catalysts prepared via SOMC that display very high metathesis activity due, in part, to the ease of product desorption. The identification of strong surface-substrate interactions that influence reactivity is particularly significant in the context of ongoing efforts to expand the scope of heterogeneous catalysis, for instance, to the upgrading of biomass feedstocks, which are often bulky and oxygenated molecules.^58,59^

## Supporting Information Available

Experimental Section, catalyst characterization, catalytic reaction data, additional solid-state NMR and FT-IR analyses, and summary of MD results (PDF)
