# Supplementary material for: Olefin-Surface Interactions: A Key Activity Parameter in Silica-Supported Olefin Metathesis Catalysts
Source: JACS Au. 2022 Mar 9;2(3):777–86. doi: 10.1021/jacsau.2c00052 (PMC8969997; doi:10.1021/jacsau.2c00052)
Supplement: Supplementary file 1 — au2c00052_si_001.pdf [file au2c00052_si_001.pdf]

Supporting information for:

Olefin-surface interactions: a key activity parameter in silica-supported olefin metathesis catalysts

*Zachariah J. Berkson,[a] Moritz Bernhardt,[a] Simon L. Schlapansky,[a] Mathis J. Benedikter,[b] Michael R. Buchmeiser,[b] Gregory A. Price,[c] Glenn J. Sunley,[c] Christophe Copéret[a]**

AUTHOR ADDRESSES

[a] Department of Chemistry and Applied Bioscience, ETH Zürich, Vladimir-Prelog-Weg 2, 8093 Zürich, Switzerland.

[b] Institute of Polymer Chemistry, Universität Stuttgart, Pfaffenwaldring 55, 70569 Stuttgart, Germany

[c] Applied Sciences, bp Innovation & Engineering, BP plc, Saltend, Hull, HU12 8DS, UK

Table of Contents

Experimental section..3

General procedures 3

Catalyst syntheses 3

Substrate preparation 5

Catalytic activity measurements 6

Solid-state NMR analyses..6

Ab initio Molecular dynamics simulations 8

FTIR Spectra...10

Catalytic reaction data...12

Analyses of post-reaction catalysts 33

Summary of MD simulation results 44

References..44

Experimental section

General procedures

All experiments were carried out under dry and oxygen-free nitrogen or argon atmosphere using Schlenk techniques or an MBraun or GS glovebox equipped with a purifier unit. Syntheses were carried out using high vacuum lines (10^{-5} mbar) and glovebox techniques. Toluene was purified using a double MBraun SPS alumina column. C_6H_6 and C_6D_6 were distilled from Na/benzophenone. 1,2-Dichlorobenzene and 1,1,2,2-tetrachloroethane were distilled from calcium hydride. Al_2O_3 was dried at 250 °C under high vacuum overnight. Molecular sieves were activated at 300 °C under high vacuum overnight. BASF Selexsorb® CD1/8''was calcined at 550 °C in air for 12 h and heated under high vacuum at 500°C for 2 h. All solvents were degassed by three consecutive freeze-pump-thaw cycles and stored over molecular sieves. The organosilicon reductant 1-methyl-3,6-bis(trimethylsilyl)-1,4-cyclohexadiene (MBTCD) was prepared following literature procedures.[1] Elemental analyses were performed at Mikroanalytisches Labor Pascher, Germany. Solution ^{1}H NMR spectra were recorded in C_6D_6 at room temperature using Bruker DRX 250 or DRX 300 NMR spectrometers and were referenced to the ^{1}H signal from residual C_6H_6 at 7.16 ppm. All infrared (IR) spectra were recorded using a Bruker α-T spectrometer in an Ar glovebox equipped with OPUS software. A typical IR measurement consisted of acquisition of 32 scans in the region from 4000 to 400 cm^{-1}.

Catalyst syntheses

$(\equiv SiO)_2Mo(=O)_2$-red was prepared based on a previously reported procedure.[2] In brief, $MoO_2[OSi(OtBu)_3]_2\cdot(thf)$ was prepared following literature procedures.[3] Amorphous fumed silica (Aerosil Degussa, 200 m^2/g) was compacted with distilled water, calcined at 500 °C under

air for at least 4 h and treated under vacuum (10^{-5} mbar) at 500 °C for 12 h and then at 700 °C for at least 12 h (ramp rate 1 °C/min), yielding dehydroxylated silica, denoted SiO_{2-700}. In a typical synthesis, a solution of 175.3 mg of $MoO_2[OSi(OtBu)_3]_2$·(thf) in benzene/1,2-dimethoxyethane (1 M dme) was slowly added to a suspension of SiO_{2-700} (0.854 g, 1.06 equiv. Si-OH) in benzene at room temperature. The suspension was slowly stirred at room temperature for 16 h. The white solid was washed 5 times with benzene (2 mL) and the resulting solid was dried thoroughly under high vacuum (10^{-5} mbar), yielding **(≡SiO)$_2$Mo(=O)$_2$[OSi(OtBu)$_3$]** as white particles. Analysis of the supernatant and the washing solution by ^{1}H solution state NMR spectroscopy using ferrocene as an internal standard indicated the consumption of 0.52 equiv of $MoO_2[OSi(OtBu)_3]_2$·(thf). Elemental analysis of the grafted compound yielded 1.47 wt% Mo. **(≡SiO)$_2$Mo(=O)$_2$[OSi(OtBu)$_3$]** was then loaded into a quartz flow reactor, placed under high vacuum (10^{-5} mbar) and heated at 200 °C for 4 h followed by 400 °C (1°C/min) for 4 h. The reactor was cooled to ambient temperature under vacuum, after which it was placed under flow of synthetic air (passed through a purifier containing activated molecular sieves) and calcined at 400 °C (1°C/min) for 4 h, yielding **(≡SiO)$_2$Mo(=O)$_2$** as white particles. The material was then evacuated under high vacuum (10^{-5} mbar) and stored in a glovebox under inert argon or nitrogen atmosphere prior to the catalytic reaction tests. Elemental analyses of separate batches of **(≡SiO)$_2$Mo(=O)$_2$** yielded 1.52-1.56 wt% Mo. IR spectra of SiO_{2-700}, **(≡SiO)$_2$Mo(=O)$_2$[OSi(OtBu)$_3$]**, and **(≡SiO)$_2$Mo(=O)$_2$** are shown in Figure S1. Immediately before catalytic reaction tests, **(≡SiO)$_2$Mo(=O)$_2$-red** was prepared by reduction of **(≡SiO)$_2$Mo(=O)$_2$** with 2 equiv. MBTCD per Mo in a benzene solution (0.08 mg/μL).

MoO$_x$@SiO$_2$-red was prepared following an incipient wetness impregnation approach based on literature procedures.[4] Amorphous fumed silica (Aerosil Degussa, 200 m^2/g) with a pore volume of ca. 1.5 cm^3/g found by N_2 adsorption was calcined under flowing synthetic air (30 standard

cubic centimeters per minute, sccm) at 500 °C to remove adsorbed organics. Freshly calcined SiO_2 (1 g) in a glass flask was impregnated with 1.5 mL/g of an aqueous solution of $(NH_4)_6Mo_7O_{24}\cdot(H_2O)_4$ in milliQ water (55 mg/mL). The $(NH_4)_6Mo_7O_{24}\cdot(H_2O)_4$ solution was added in 100 μL increments while stirring with a glass stir rod. The impregnated silica was allowed to dry overnight under ambient conditions, then loaded into a glass flow reactor and heated under flowing synthetic air to 80 °C for 4 h, 110 °C for 4 h, and finally calcined at 500 °C for 4 h (ramp rate 1 °C/min.). The resultant material (**MoO_x@SiO_2**) was then evacuated under high vacuum (10^{-5} mbar) and stored in a glovebox under inert argon or nitrogen atmosphere prior to the catalytic reaction tests. Elemental analysis of **MoO_x@SiO_2** yielded 3.52 wt% Mo. The IR spectrum of **MoO_x@SiO_2** is shown in Figure S2. Immediately before catalytic reaction tests, **MoO_x@SiO_2-red** was prepared by reduction of **MoO_x@SiO_2** with 2 equiv. MBTCD per Mo in a benzene solution (0.08 mg/μL).

The cationic Mo alkylidenes **Mo^+** and **Mo^+/$SiO_{2\text{-}700}$** were synthesized as previously reported.[5,6]

Substrate preparation

1-Octene (>95%), 1-nonene (>95%), 1-tridecene (>95%), 1-hexadecene (>95%), and 1-eicosene (>95%) were obtained from Tokyo Chemical Industry (TCI). Decalin (98%) was obtained from Acros Organics for use as an internal standard. All substrates and decalin were rigorously purified[7] by drying overnight over sodium metal, distillation *in vacuo*, degassing by at least three freeze-pump-thaw cycles, and storage in the glovebox. Before the preparation of the olefin stock solutions, the neat olefins were stored over BASF Selexsorb® for 4 h and filtered 3x through a pad of activated alumina. After this purification protocol the substrates were found to be typically ca. 97% pure by GC, with the impurities being predominantly internal isomers of the terminal olefins.

Note that prolonged exposure to Selexsorb or molecular sieves promotes isomerization of the terminal olefins to their internal isomers.[7] Decalin was passed 3 times through a pad of activated alumina and stored over Selexsorb. 1 M stock solutions of the terminal olefins in 1,2-dichlorobenzene with 0.1 M decalin as an internal standard were prepared before the catalytic tests and used immediately.

Catalytic activity measurements

The terminal olefin metathesis reactions were conducted in a nitrogen-filled glovebox using the High-Throughput Experimentation facility at ETH Zürich (HTE@ETH). The catalysts were weighed out into 10 mL reaction vials, heated to the reaction temperature (30 or 70 °C), and shaken at 300 rpm. The reaction vials were either left open over the course of the reaction to allow for ethylene removal or closed with a Teflon plate. 30 μL aliquots of the reaction mixture were taken at the specified time points, diluted with 600 μL toluene, and analyzed by GC/FID. Conversions were calculated from substrate concentration, while product formation rates were calculated from the concentrations of the metathesis products.

Preparation of post-reaction catalysts for spectroscopic analysis

After reaction of **$(\equiv SiO)_2Mo(=O)_2$-red** of **$Mo^+/SiO_{2\text{-}700}$** with 1-hexadecene or 1-nonene for the specified reaction time at 30 °C, the reaction solution was filtered off and the post-reaction catalyst was washed with benzene (3x 1 mL), dried overnight under high vacuum ($<10^{-5}$ mbar), and transferred into a glovebox where the dried material was powdered and packed into an NMR rotor.

Preparation of olefin-adsorbed $SiO_{2\text{-}700}$

The adsorption of olefins on silica devoid of Mo centers was also tested by contacting dehydroxylated silica $SiO_{2\text{-}700}$ with olefin solution for 3 h, after which the material was filtered

from the olefin solution, washed with benzene (3x 1 mL), and dried overnight under high vacuum (<10^{-5} mbar). The olefin solution was either 1 M 1-hexadecene or 0.35 M 15-triacontene, the product of 1-hexadecene metathesis which was purified by vacuum distillation (est. >95% purity by GC).

Solid-state NMR analyses

The 1D and 2D solid-state ^{1}H and ^{1}H{^{13}C} NMR spectra were acquired on a 700 MHz (16.4 T) Bruker NMR spectrometer equipped with a 1.3 mm HX MAS probe head and operating at Larmor frequencies of 176.061 and 700.135 MHz for ^{13}C and ^{1}H, respectively. The rotors were transferred to the NMR spectrometer in a tightly sealed vial with a screw-cap top, rapidly inserted into the MAS probe head, and spun with dry N_2 gas. MAS rates of 50 kHz were used with relaxation delays of 3.5 s, which was ca. 1.4 times the longest measured ^{1}H spin-lattice T_1 relaxation time (measured by ^{1}H saturation recovery). The measurements used ^{1}H $\pi/2$ pulse lengths of 2 μs (125 kHz) and ^{13}C $\pi/2$ pulse lengths of 3 μs (83 kHz). The 2D ^{1}H{^{13}C} correlation spectrum was acquired using a 2D dipolar-mediated HMQC sequence with 60 rotor periods of $SR4$ recoupling[8] to reintroduce the ^{1}H-^{13}C dipole-dipole couplings. 64 t_1 increments were used in the indirect dimension and 480 transients for a total acquisition time of 30 h. ^{1}H spin-spin T_2 relaxation times were measured using a pseudo-2D CPMG sequence[9] with varied lengths (τ) of rotor-synchronized echo trains.

The 1D and 2D ^{13}C{^{1}H} and ^{29}Si{^{1}H} DNP-NMR spectra were acquired on a Bruker Avance III 600 MHz (14.1 T) DNP NMR spectrometer equipped with a low-temperature 3.2 mm triple-resonance MAS cryo-probe operating at Larmor frequencies of 599.900, 150.858, 119.166 MHz for ^{1}H, ^{13}C, and ^{29}Si, respectively. For the DNP-enhanced NMR measurements, **($\equiv$SiO)$_2$Mo(=O)$_2$-red** was reacted with 1-hexadecene for 4 h, washed and dried as described above, and transferred

into a glovebox where the dried material was ground and impregnated with a 16 mM solution of TEKPol biradical[10] in 1,1,2,2-tetrachloroethane. The material was then packed into a sapphire MAS NMR rotor with a zirconia cap, transferred to the NMR spectrometer in a tightly sealed vial with a screw-cap top and rapidly inserted into the pre-cooled (100 K) MAS NMR probe head. DNP-NMR measurements were acquired at 10 kHz MAS under continuous microwave irradiation at 395 GHz. The microwave on/off DNP enhancements were measured by comparison of the $^{13}C\{^{1}H\}$ CPMAS spectra acquired with and without microwave irradiation and were found to be ca. 4 for the ^{13}C signals associated with surface-bound organic species. For all DNP-NMR measurements, a relaxation delay of 3 s was used. The 2D $^{29}Si\{^{1}H\}$ DNP-HETCOR spectra in Figures 5 and S20 were acquired with ^{29}Si-^{1}H contact times of 0.5 or 5 ms, 512 transients, and 16 or 30 t_1 increments in the indirect dimension for total acquisition times of 7 or 13 h, respectively. The 2D $^{13}C\{^{1}H\}$ DNP-HETCOR spectrum in Figure S19 was acquired with a ^{13}C-^{1}H contact times of 2 ms, 64 transients, and 64 t_1 increments in the indirect dimension for total acquisition time of 3.5 h. All of the 2D DNP-NMR spectra were acquired with 100 kHz eDUMBO homonuclear decoupling[11] in the indirect ^{1}H dimension and 100 kHz SPINAL-64 heteronuclear ^{1}H decoupling[12] during the acquisition period.

Ab initio Molecular dynamics simulations

Olefin geometries were pre-optimized using the hybrid functional B3LYP[13–17] in combination with the 6-31g(d) basis set[18] in Gaussian 09 (revision d1).[19] Before ab initio molecular dynamics (AIMD) were run the structures were optimized using the conjugated gradient method (convergence criteria) in the CP2K (3.0) program.[20] The revPBE[21–23] functional including Grimme dispersion correction[24] and Becke-Johnson damping functions (D3-BJ)[24,25] was used in combination with the double-zeta (DZVP-MOLOPT-SR-GTH)[26] basis set and the associated

GTH pseudopotential.[27] For the AIMD calculation, the olefin was placed on a previously optimized silica surface model (cell size 21.4 Å x 21.4 Å x 34.2 Å)[28] and the system was optimized using the same level of theory.

The system was then equilibrated at 295 K in the NVT ensemble using a 1 fs for each of the 3000 steps and the temperature was controlled using a 295 K canonical sampling through velocity rescaling (CSVR) thermostat [29] (time step 100 fs). All hydrogen atoms were substituted by deuterium ones in order to allow a longer time step and to prevent artificial bond breaking due to the fast motion of H atoms. Furthermore, a weak external potential of the form $1*10^{-12}*((Z-12)^2)^4$ was added to disfavor desorption of the olefin from the surface. The four lowest energy structures for each olefin found by AIMD were again optimized with the same level of theory in CP2K to obtain optimized geometries of the olefins at the surface. From these four geometries the values in Table S7 were extracted.

FTIR Spectra of as-prepared (pre)catalysts

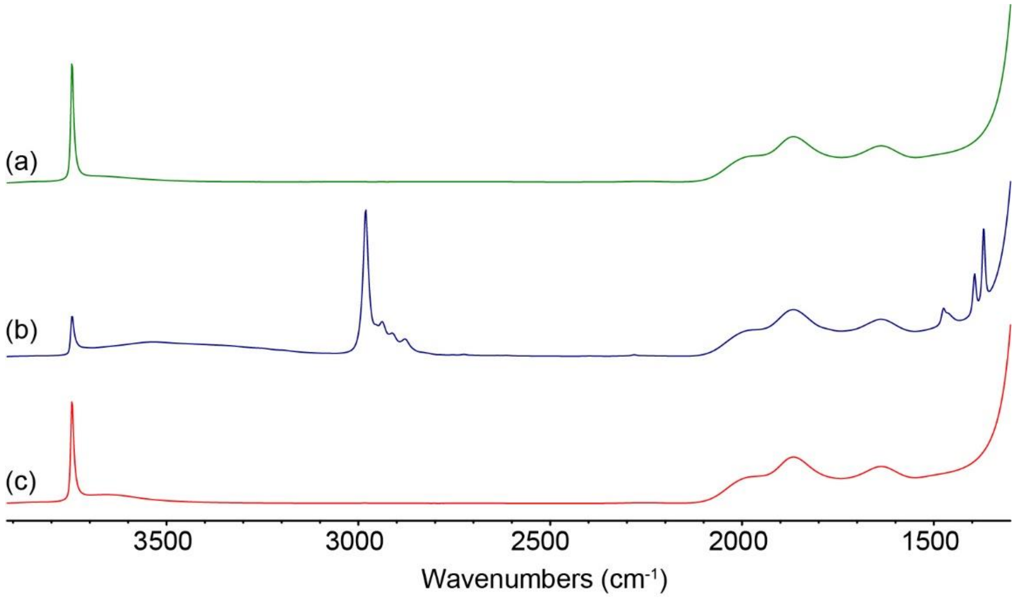

Figure S1. Transmission FTIR spectra of (a) $SiO_{2\text{-}700}$, (b) grafted molecular Mo dioxo siloxide **$(\equiv SiO)_2Mo(=O)_2[OSi(OtBu)_3]$**, and (c) **$(\equiv SiO)_2Mo(O)_2$**.

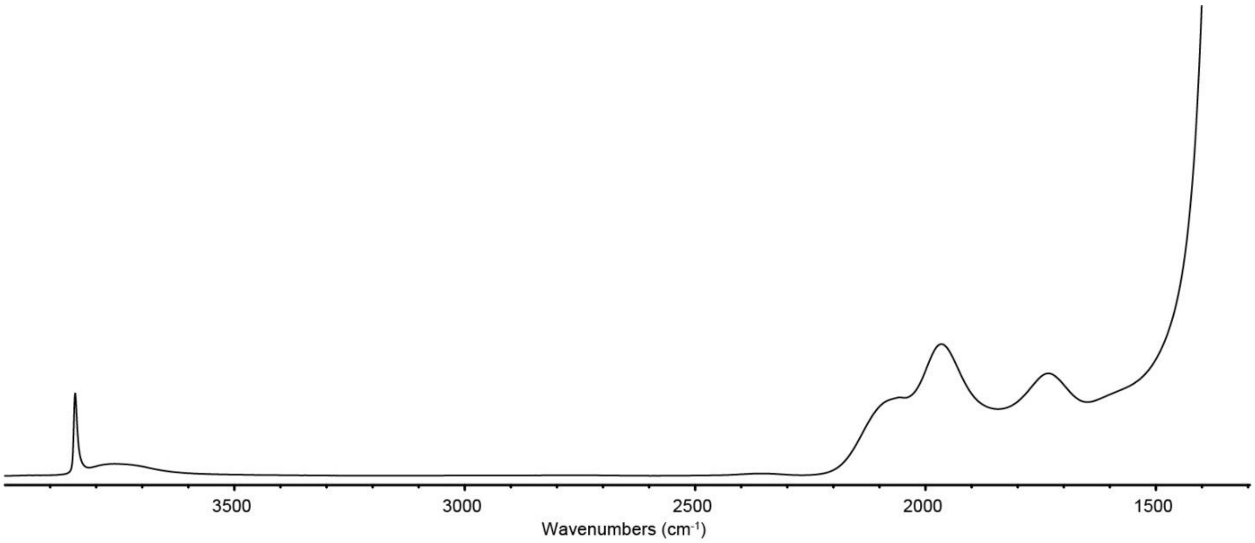

Figure S2. Transmission FTIR spectrum of **MoO_x/SiO_2**.

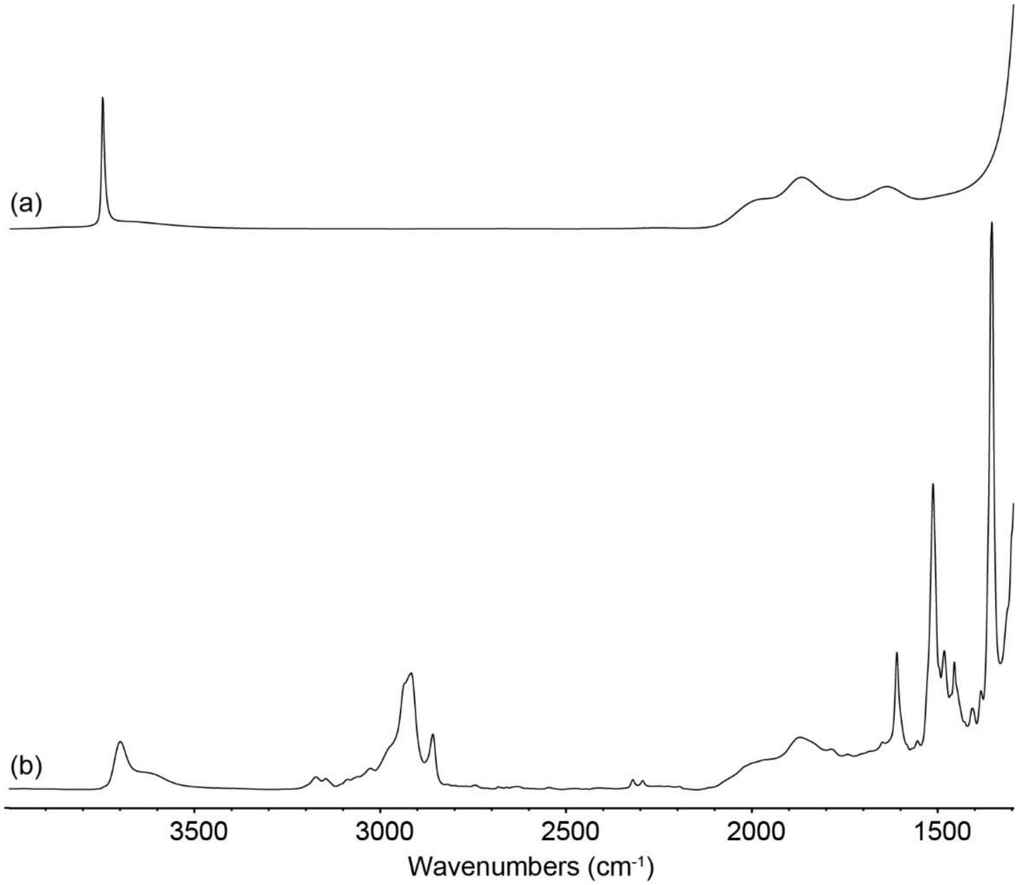

Figure S3. Transmission FTIR spectra of (a) $SiO_{2\text{-}700}$ and (b) **Mo^{+}/SiO_2.**

Catalytic reaction data

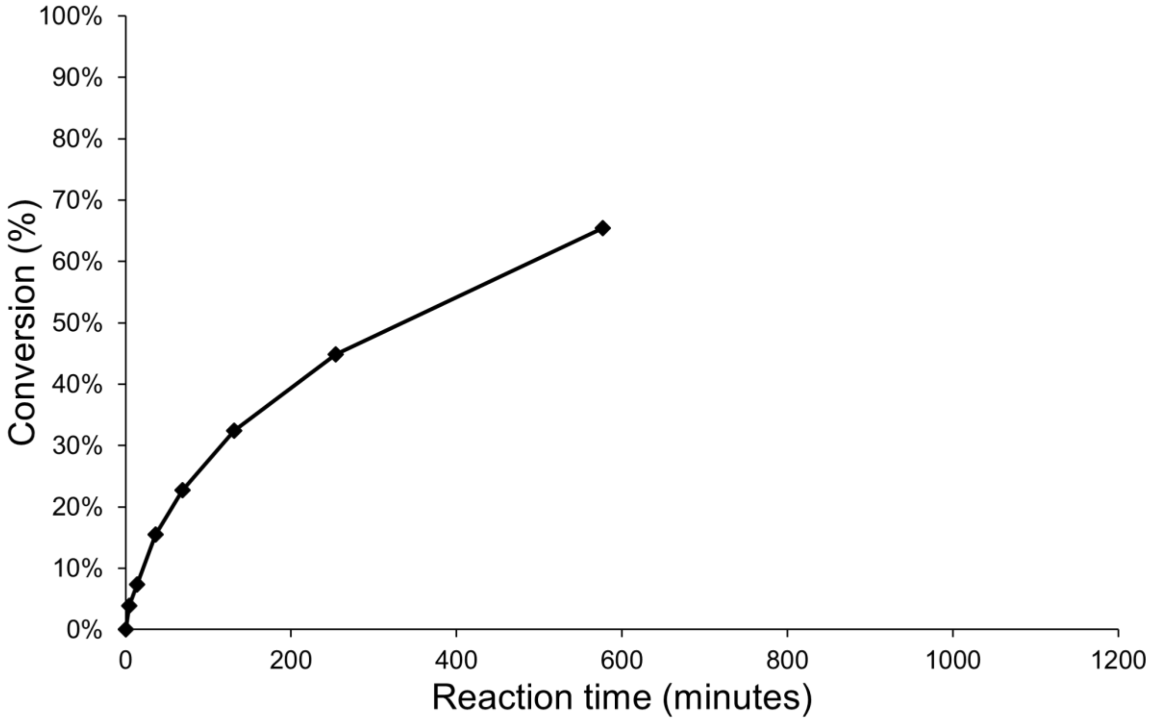

Figure S4. Metathesis of 1-nonene by **(≡SiO)$_2$Mo(=O)$_2$-red** at 70 °C. Open reaction vial, ca. 0.1 mol% Mo.

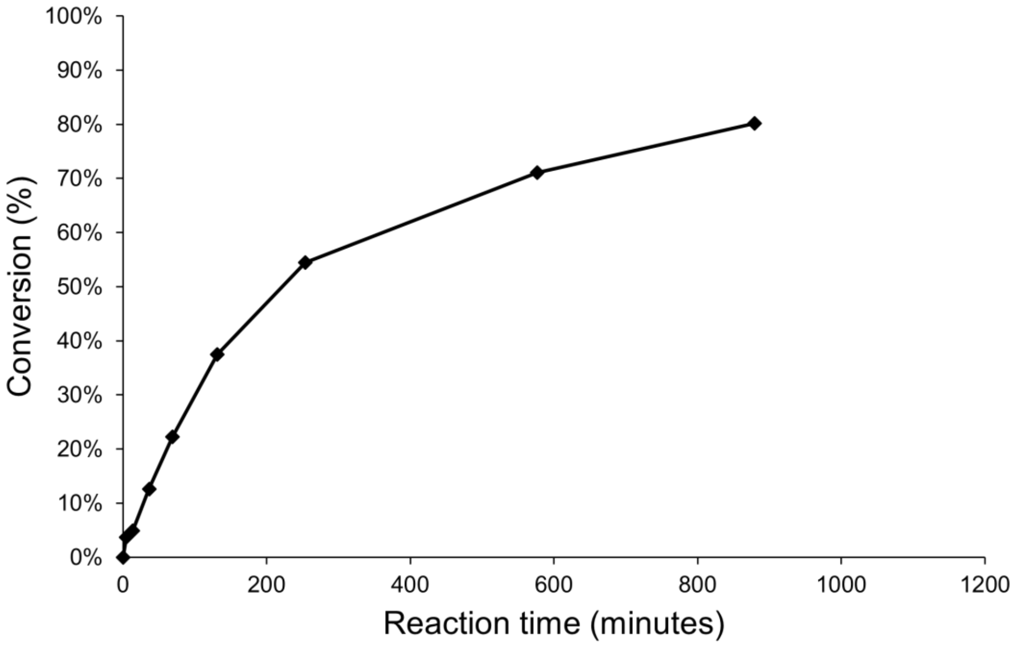

Figure S5. Metathesis of 1-nonene by **(≡SiO)$_2$Mo(=O)$_2$-red** at 30 °C. Open reaction vial, ca. 0.1 mol% Mo.

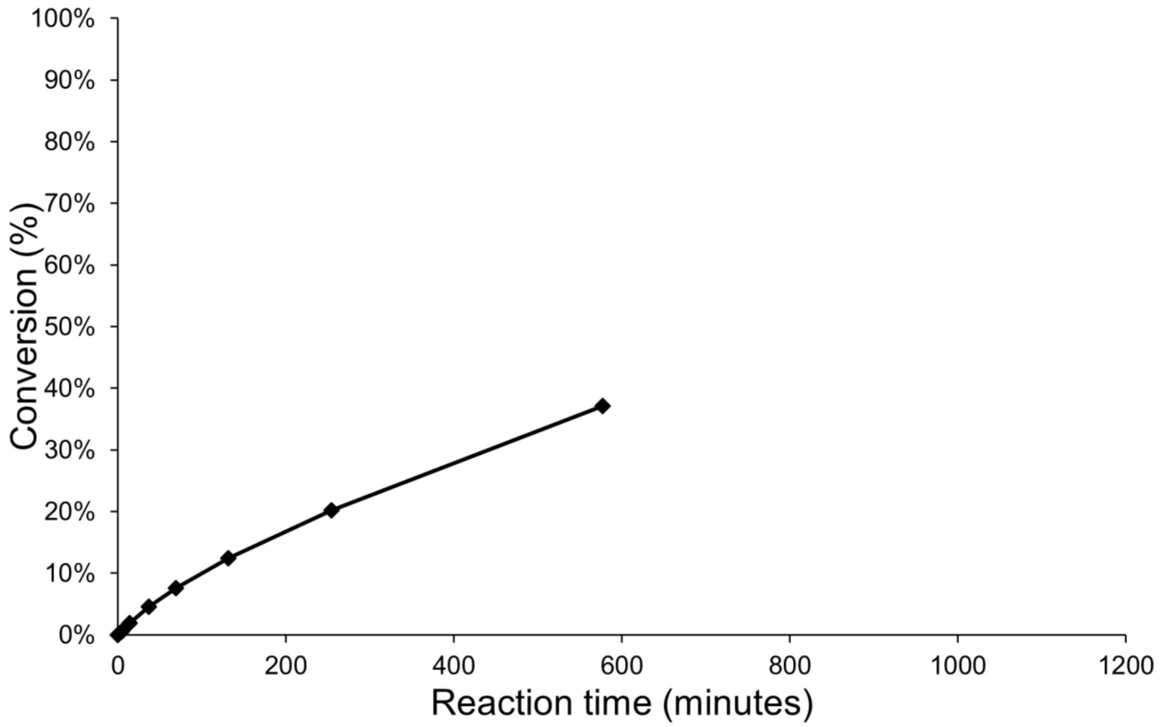

Figure S6. Metathesis of 1-nonene by **(≡SiO)$_2$W(=O)$_2$-red** at 70 °C. Open reaction vial, ca. 0.1 mol% W.

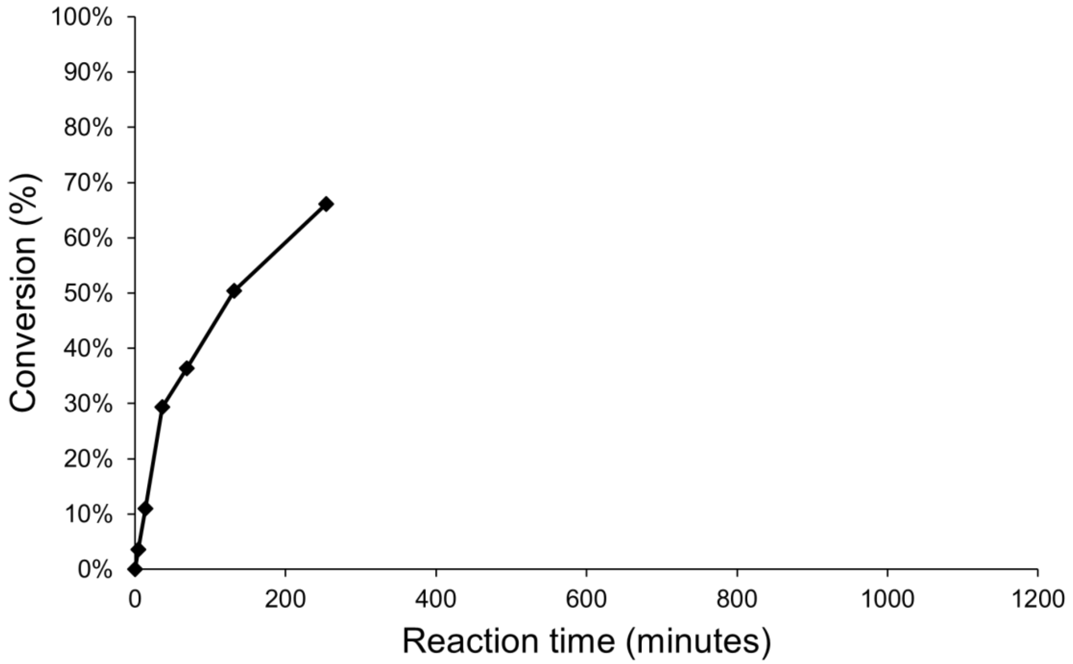

Figure S7. Metathesis of 1-nonene by **MoO$_x$/SiO$_2$-red** at 70 °C. Open reaction vial, ca. 0.1 mol% Mo.

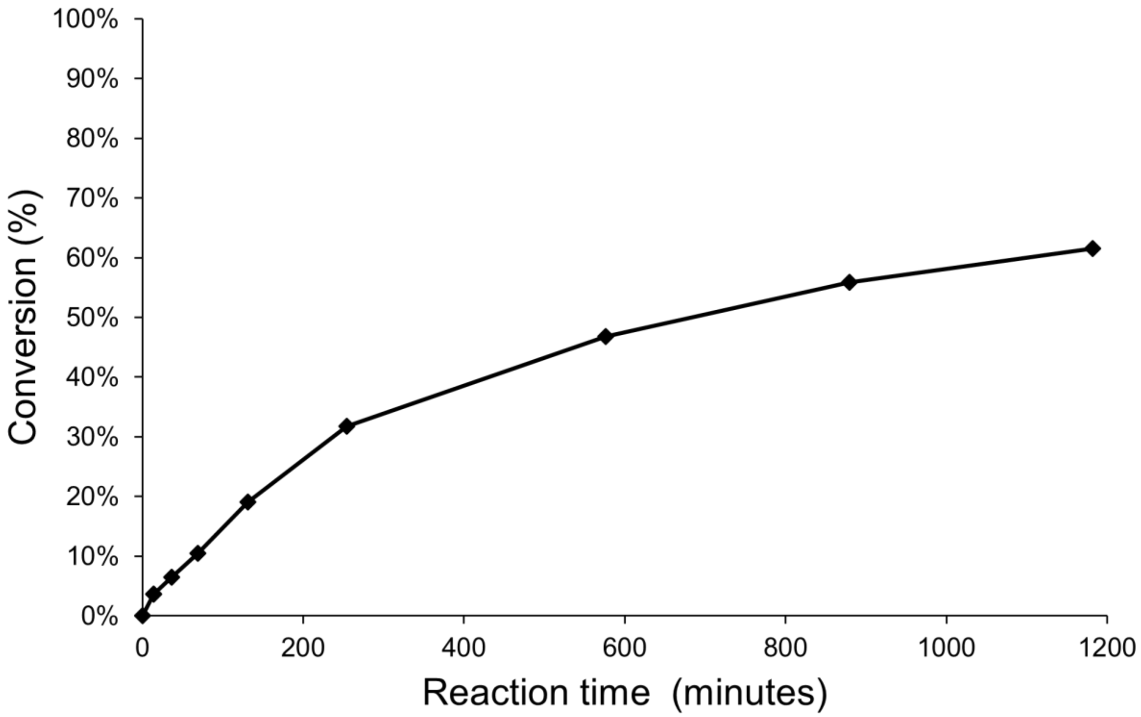

Figure S8. Metathesis of 1-nonene by **MoO_x/SiO_2-red** at 30 °C. Open reaction vial, ca. 0.1 mol% Mo.

Table S1. Summary of catalytic data for metathesis of 1-nonene by **(≡SiO)$_2$Mo(=O)$_2$-red**, **(≡SiO)$_2$W(=O)$_2$-red**, and **MoO$_x$/SiO$_2$-red**.[a]

**Catalyst**	**Temperature (°C)**	**Maximum product formation rate (mmol product/[mmol Mo min])**[b]	**Initial product E/Z ratio**[b]	**Substrate conversion after 4 h (%)**	**Selectivity for olefin metathesis product (%)**
**(≡SiO)$_2$Mo(=O)$_2$-red**	70	2.6	2.11	45	89
**(≡SiO)$_2$Mo(=O)$_2$-red**	30	2.6	2.34	54	99
**(≡SiO)$_2$W(=O)$_2$-red**	70	0.7	1.45	20	99
**MoO$_x$/SiO$_2$-red**	70	3.9	2.07	66	94
**MoO$_x$/SiO$_2$-red**	30	0.9	2.35	32	98

[a] Conditions: 70 or 30 °C, open reaction vials, 2.5 mL reaction volume, substrate : Mo = 1000 : 1 (ca. 0.1 mol% Mo)

[b] Measured between 10 and 13 minutes reaction time for each catalyst.

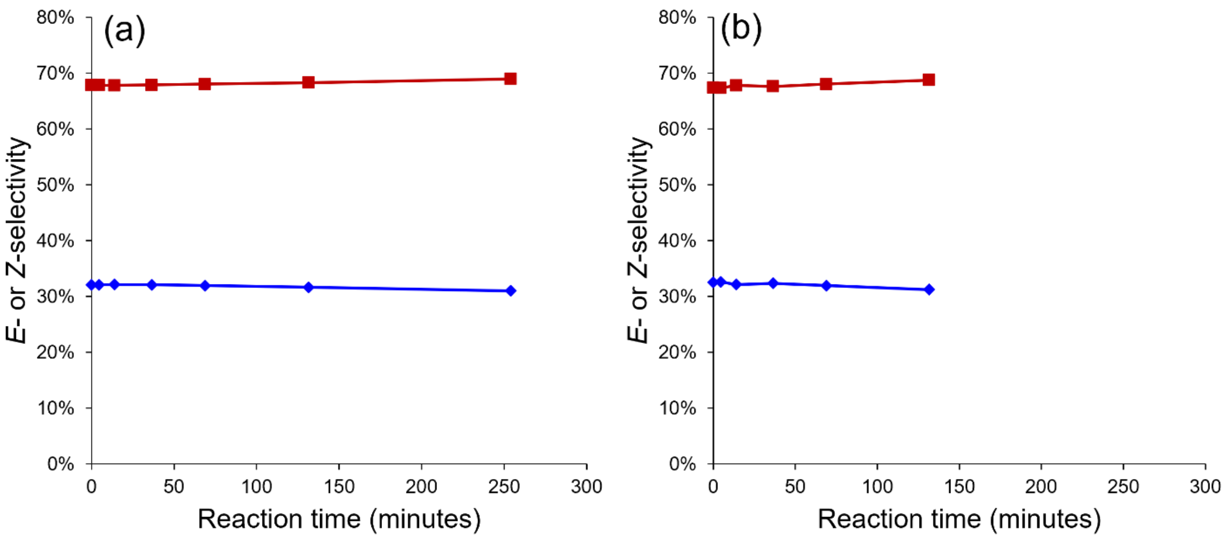

Figure S9. 1-nonene metathesis product E (red) and Z (blue) selectivity for (a) **(≡SiO)$_2$Mo(=O)$_2$-red** and (b) **MoO$_x$/SiO$_2$-red** at 70 °C.

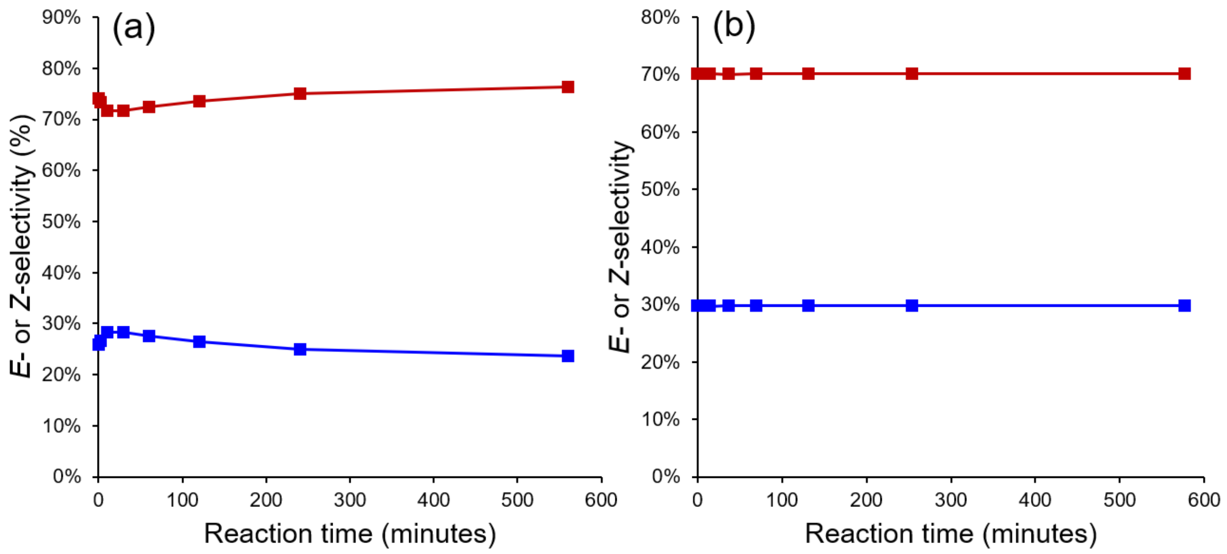

Figure S10. 1-nonene metathesis product E (red) and Z (blue) selectivity for (a) **(≡SiO)$_2$Mo(=O)$_2$-red** and (b) **MoO$_x$/SiO$_2$-red** at 30 °C.

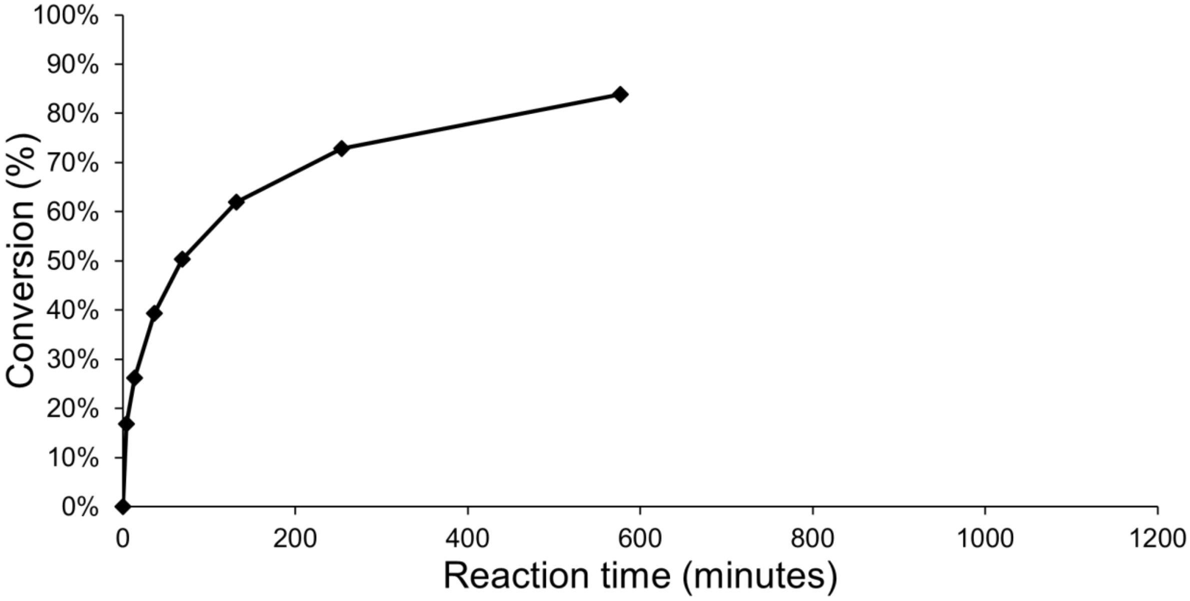

Figure S11. Metathesis of 1-octene by **(≡SiO)$_2$Mo(=O)$_2$-red** at 70 °C. Closed reaction vial, ca. 0.1 mol% Mo.

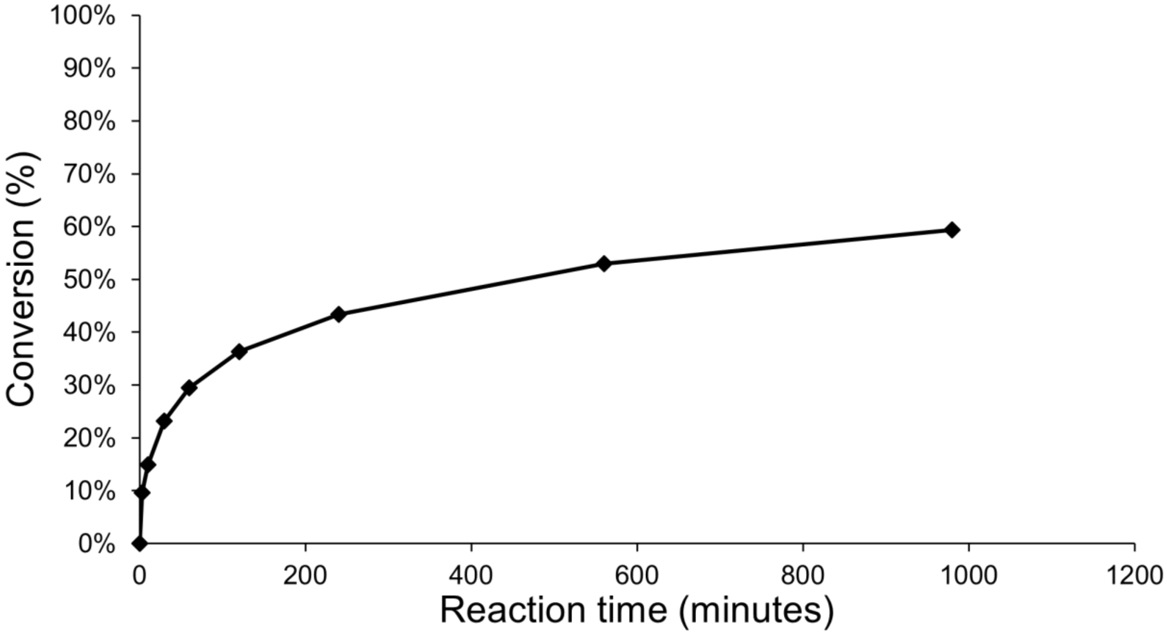

Figure S12. Metathesis of 1-nonene by **(≡SiO)$_2$Mo(=O)$_2$-red** at 70 °C. Closed reaction vial, ca. 0.1 mol% Mo.

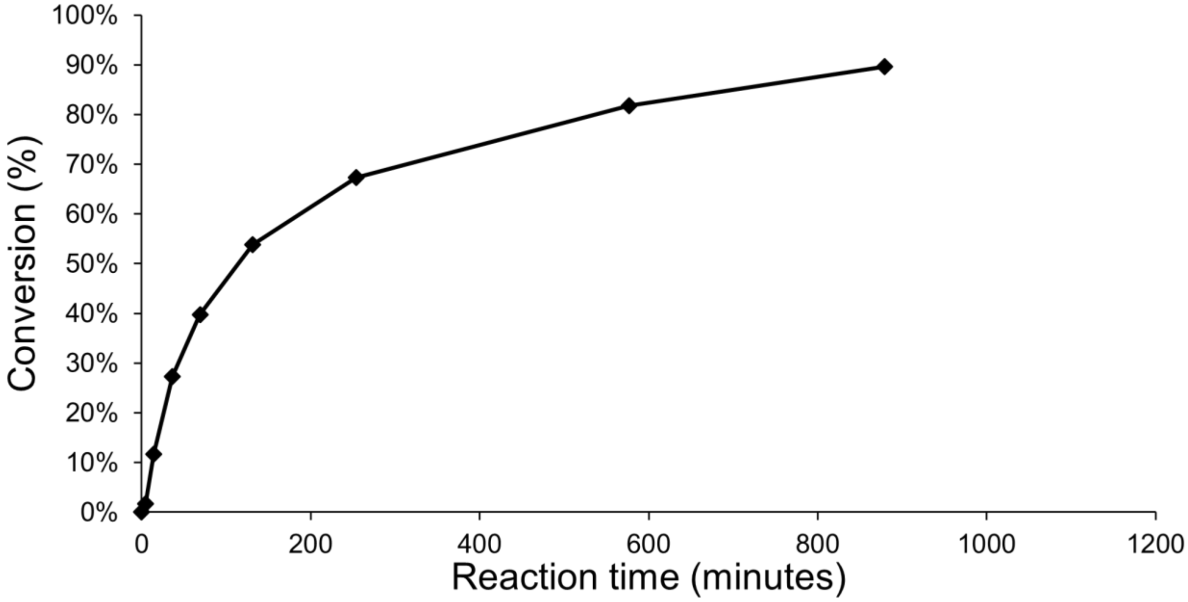

Figure S13. Metathesis of 1-tridecene by **(≡SiO)$_2$Mo(=O)$_2$-red** at 70 °C. Closed reaction vial, ca. 0.1 mol% Mo.

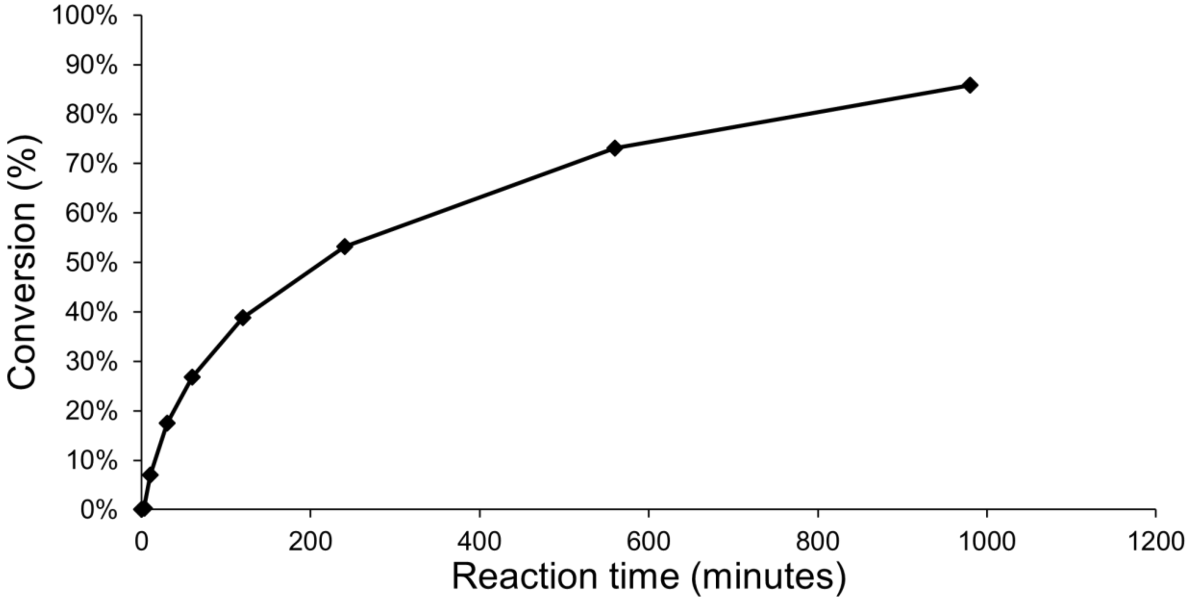

Figure S14. Metathesis of 1-hexadecene by **(≡SiO)$_2$Mo(=O)$_2$-red** at 70 °C. Closed reaction vial, ca. 0.1 mol% Mo.

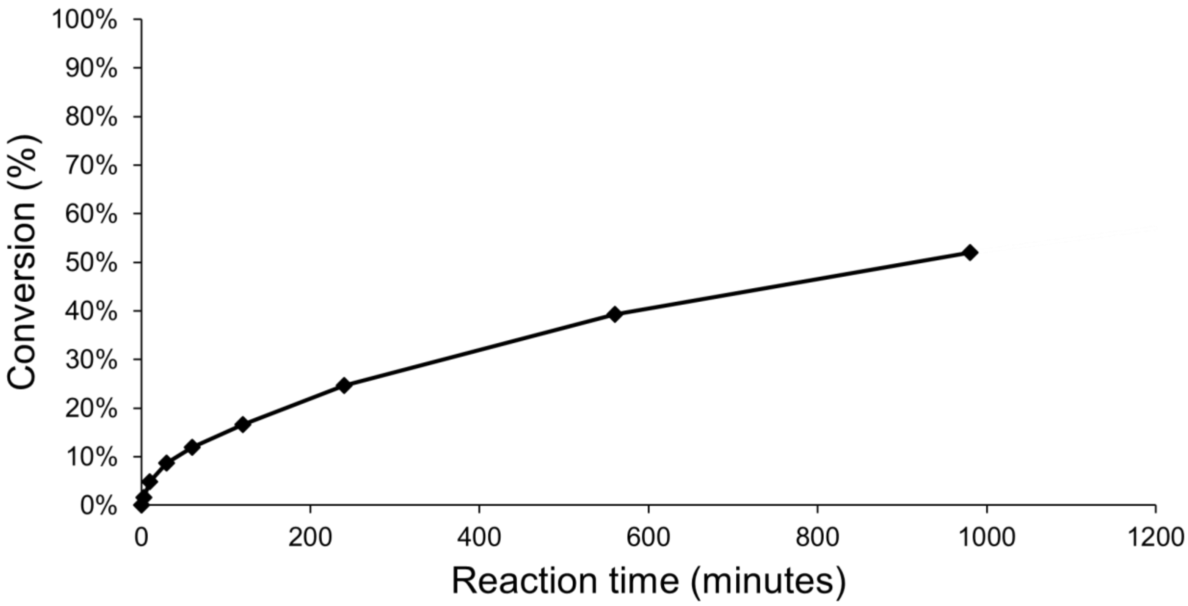

Figure S15. Metathesis of 1-eicosene by **(≡SiO)$_2$Mo(=O)$_2$-red** at 70 °C. Closed reaction vial, ca. 0.2 mol% Mo.

Table S2. Summary of catalytic data for metathesis of linear α-olefins by **(≡SiO)$_2$Mo(=O)$_2$-red** at 70 °C.[a]

**Substrate**	**Maximum product formation rate (mmol product/[mmol Mo min])**[b]	**Initial product E/Z ratio**[b]	**Substrate conversion after 4 h (%)**	**Selectivity for olefin metathesis product (%)**
1-octene	9.9	2.36	78	85
1-nonene	8.1	2.41	43	97
1-tridecane	4.5	2.33	67	96
1-hexadecane	2.7	2.30	69	91
1-eicosene	0.9	-[c]	25	-[c]

[a] Conditions: 70 °C, closed reaction vials, 2.5 mL reaction volume, substrate : Mo = 1000 : 1 (ca. 0.1 mol% Mo) for 1-octene, 1-nonene, 1-tridecene, and 1-hexadecene, substrate : Mo = 500 : 1 (ca. 0.2 mol% Mo) for 1-eicosene
[b] Measured between 10 and 13 minutes reaction time for each catalyst
[c] Product peaks not resolved

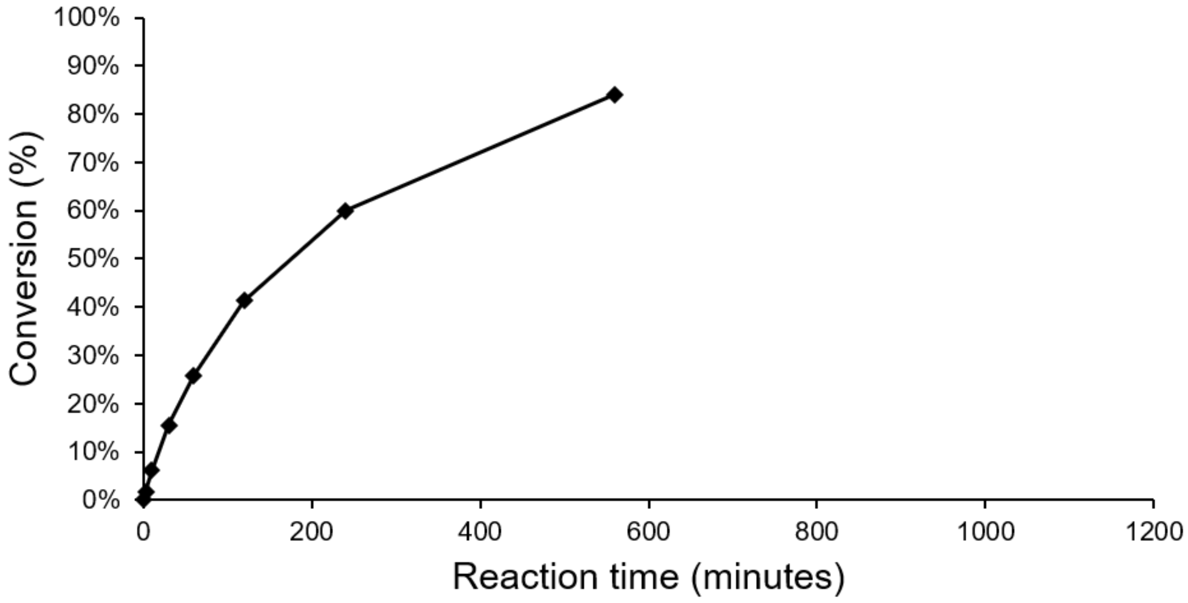

Figure S16. Metathesis of 1-octene by **(≡SiO)$_2$Mo(=O)$_2$-red** at 30 °C. Open reaction vial, ca. 0.1 mol% Mo.

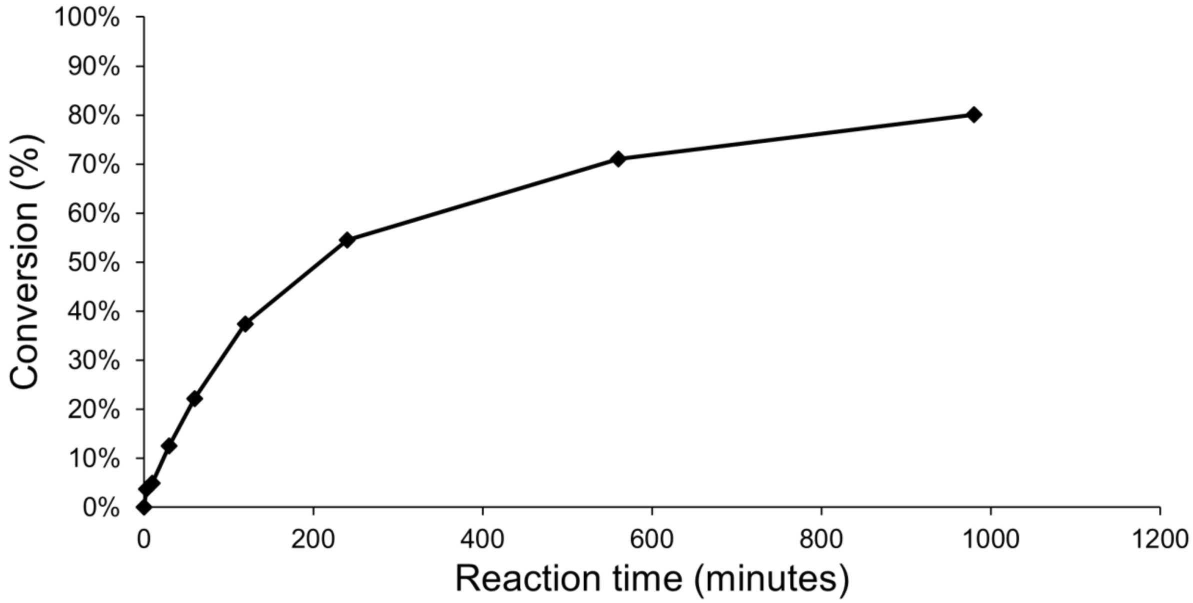

Figure S17. Metathesis of 1-nonene by **(≡SiO)$_2$Mo(=O)$_2$-red** at 30 °C. Open reaction vial, ca. 0.1 mol% Mo.

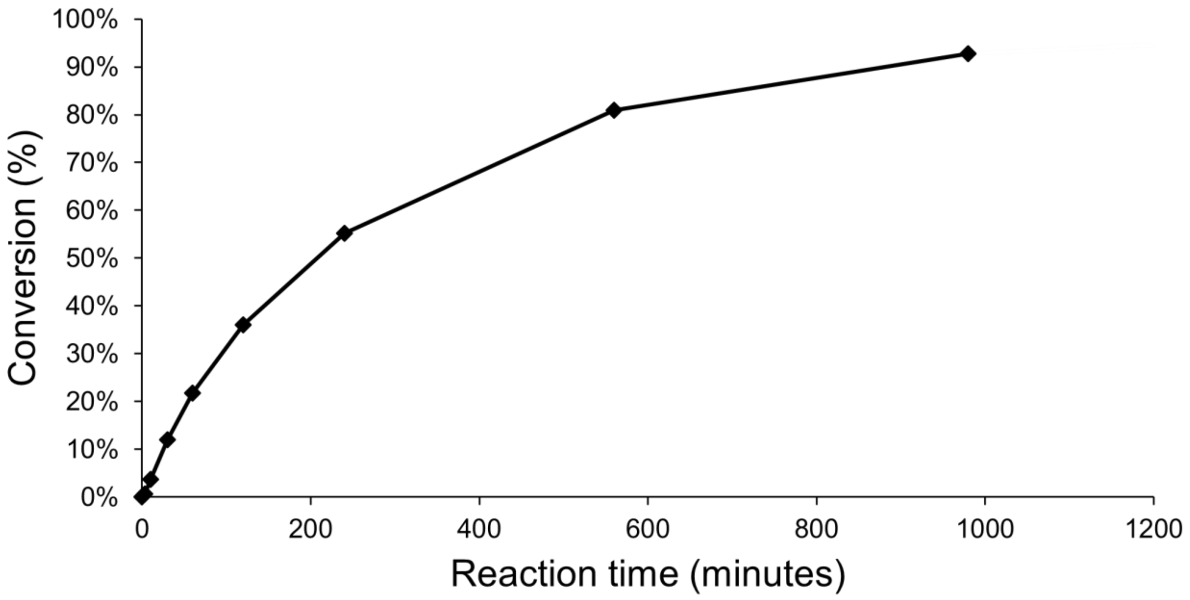

Figure S18. Metathesis of 1-tridecene by **(≡SiO)$_2$Mo(=O)$_2$-red** at 30 °C. Open reaction vial, ca. 0.1 mol% Mo.

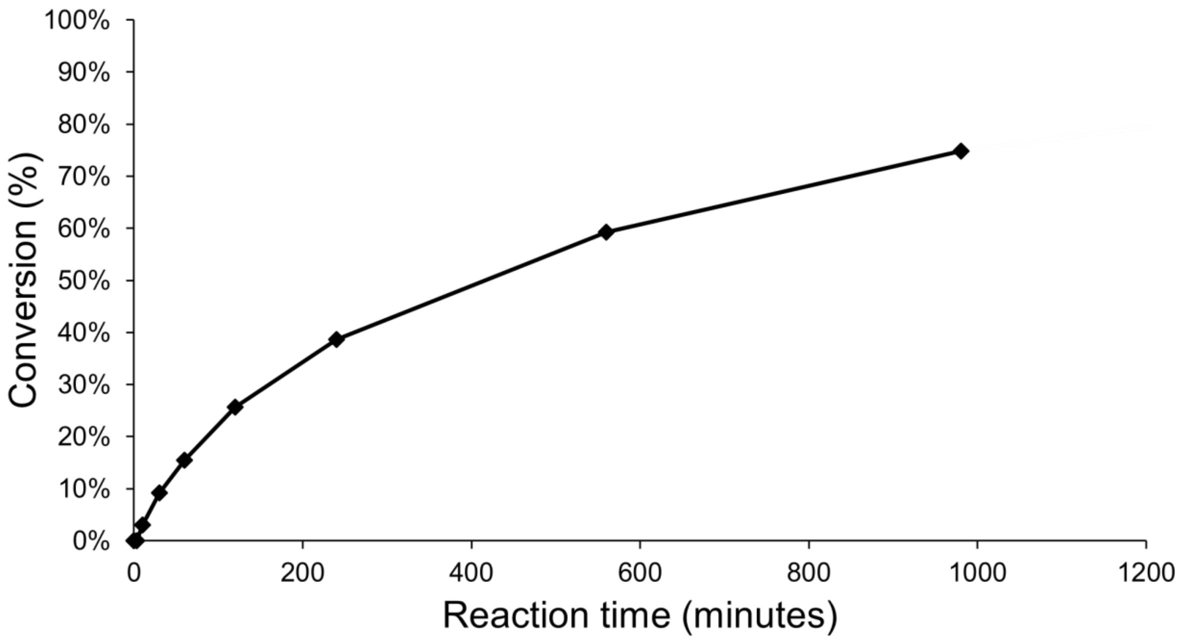

Figure S19. Metathesis of 1-hexadecene activity of **(≡SiO)$_2$Mo(=O)$_2$-red** at 30 °C. Open reaction vial, ca. 0.1 mol% Mo.

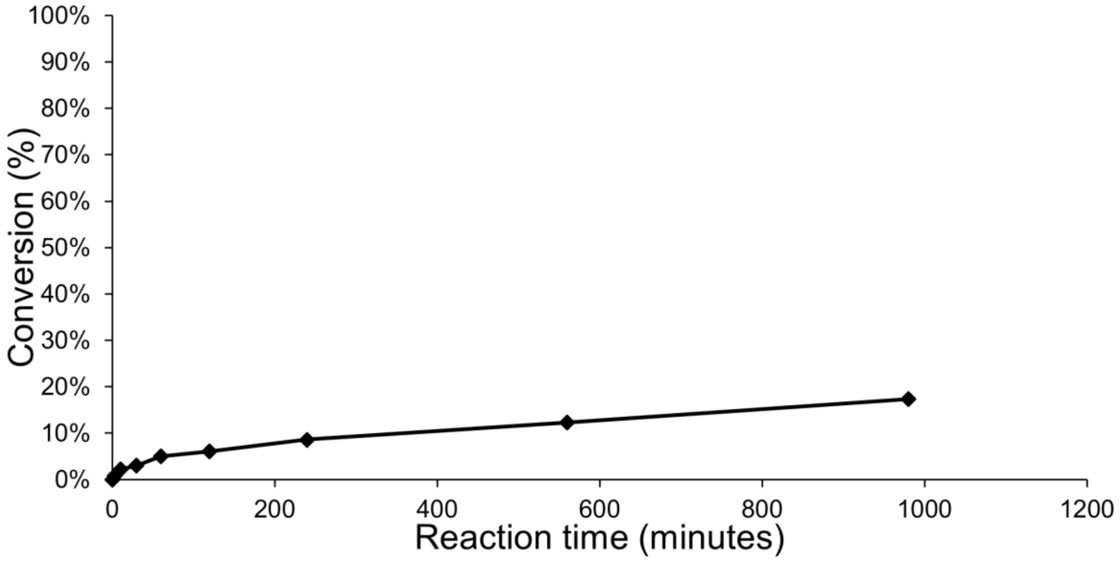

Figure S20. Metathesis of 1-eicosene by **(≡SiO)$_2$Mo(=O)$_2$-red** at 30 °C. Open reaction vial, ca. 0.2 mol% Mo.

Table S3. Summary of catalytic data for metathesis of linear α-olefins by **(≡SiO)$_2$Mo(=O)$_2$-red** at 30 °C.[a]

**Substrate**	**Maximum product formation rate (mmol product/[mmol Mo min])[b]**	**Initial product E/Z ratio[b]**	**Substrate conversion after 4 h (%)**	**Selectivity for olefin metathesis product (%)**
1-octene	3.2	2.50	60	98
1-nonene	2.6	2.34	54	99
1-tridecane	2.0	2.33	55	99
1-hexadecane	1.6	2.43	39	97
1-eicosene	0.6	-[c]	9	-[c]

[a] Conditions: 30 °C, open reaction vials, 2.5 mL reaction volume, substrate : Mo = 1000 : 1 (ca. 0.1 mol% Mo) for 1-octene, 1-nonene, 1-tridecene, and 1-hexadecene, substrate : Mo = 500 : 1 (ca. 0.2 mol% Mo) for 1-eicosene
[b] Measured between 10 and 13 minutes reaction time for each catalyst
[c] Product peaks not resolved

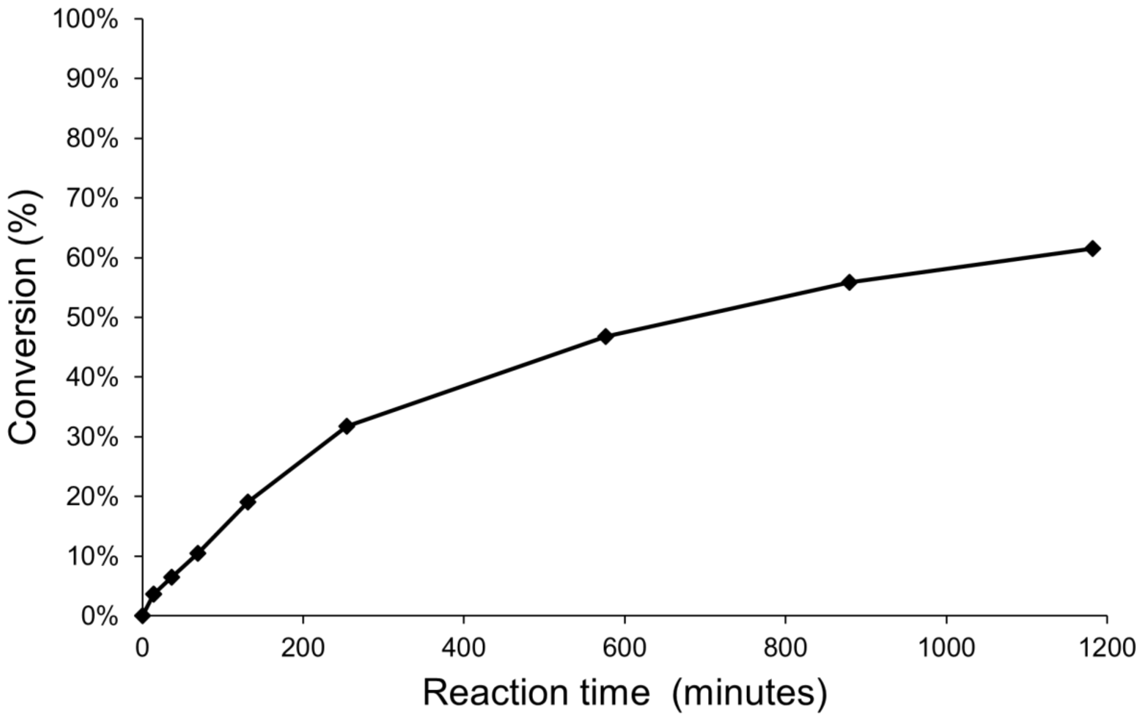

Figure S21. Metathesis of 1-nonene by **MoO_x/SiO_2-red** at 30 °C. Open reaction vial, ca. 0.1 mol% Mo.

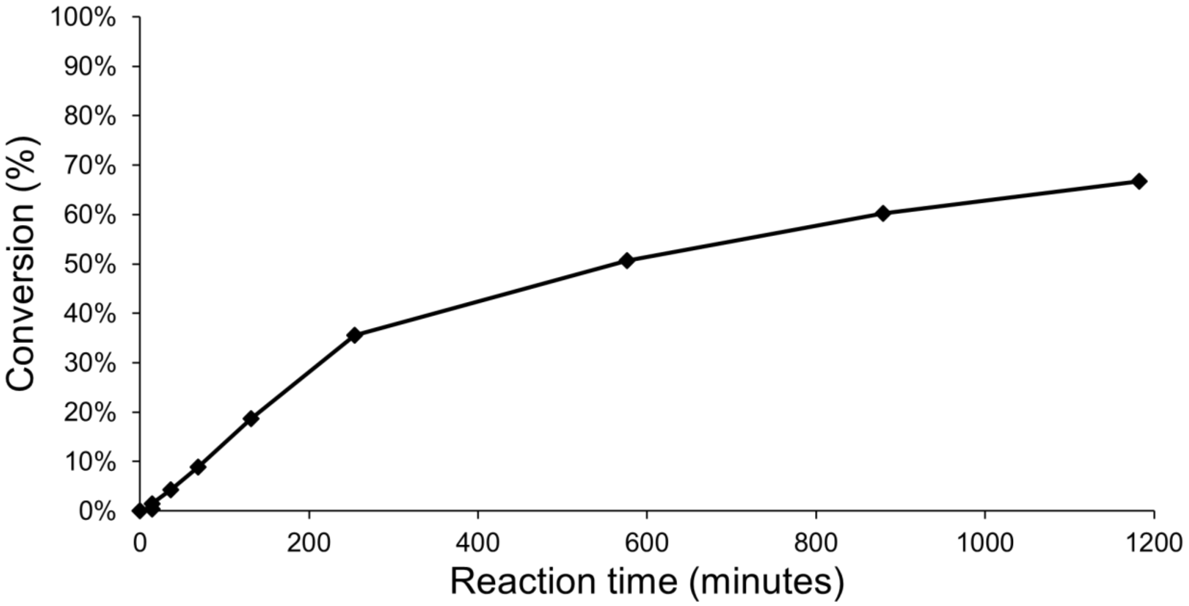

Figure S22. Metathesis of 1-tridecene by **MoO_x/SiO_2-red** at 30 °C. Open reaction vial, ca. 0.1 mol% Mo.

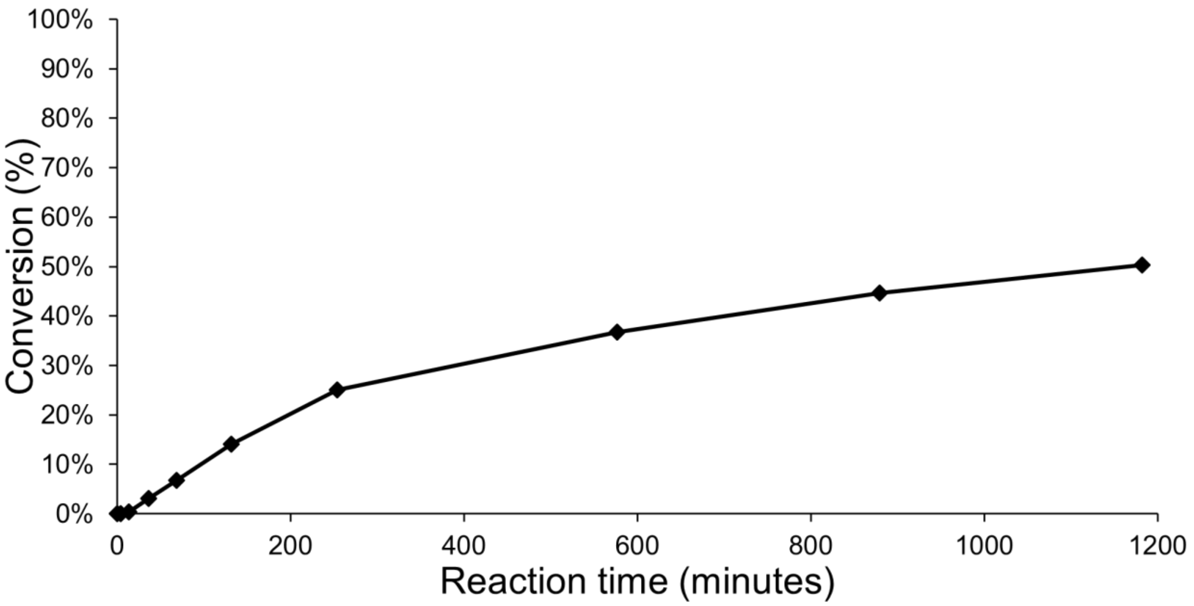

Figure S23. Metathesis of 1-hexadecene by **MoO_x/SiO_2-red** at 30 °C. Open reaction vial, ca. 0.1 mol% Mo.

Table S4. Summary of catalytic data for metathesis of linear α-olefins by **MoO_x/SiO_2-red** at 30 °C.[a]

Substrate	Maximum product formation rate (mmol product/[mmol Mo min]) at the indicated reaction time	Initial product E/Z ratio[b]	Substrate conversion after 4 h (%)	Selectivity for olefin metathesis product (%)
1-nonene	0.9 (14 minutes)	2.35	32	98
1-tridecane	0.7 (60 minutes)	2.29	33	98
1-hexadecane	0.5 (60 minutes)	2.30	25	99

[a] Conditions: 30 °C, open reaction vials, 2.5 mL reaction volume, substrate : Mo = 1000 : 1 (ca. 0.1 mol% Mo)
[b] Measured after 10-14 minutes of reaction time

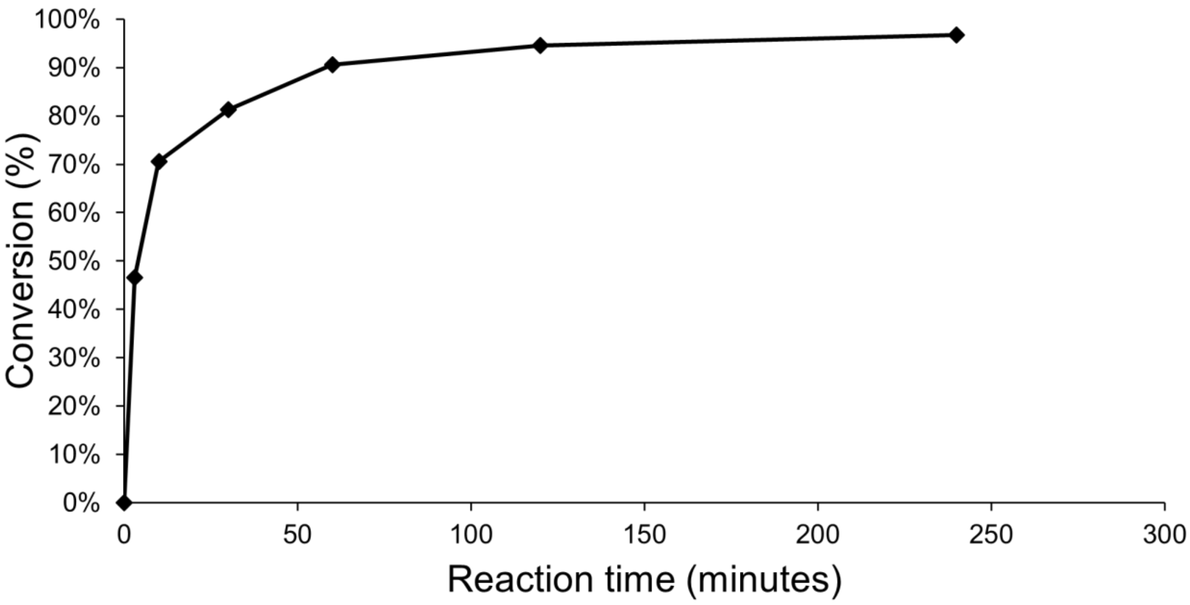

Figure S24. Metathesis of 1-octene by **Mo**$^+$ at 30 °C. Open reaction vial, ca. 0.02 mol% Mo.

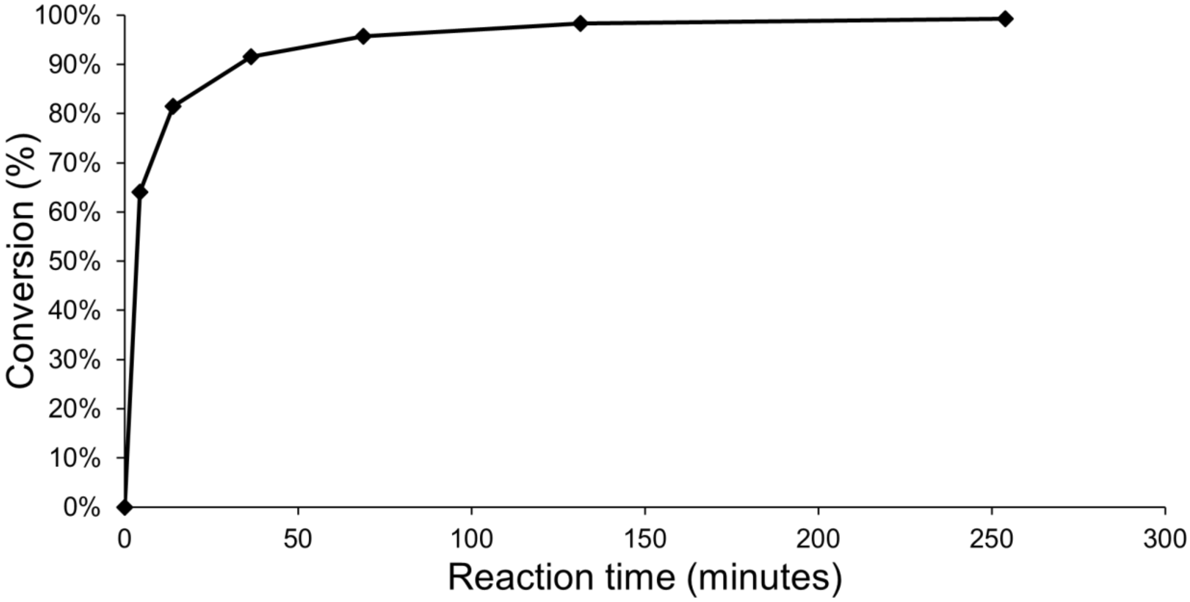

Figure S25. Metathesis of 1-nonene by **Mo**$^+$ at 30 °C. Open reaction vial, ca. 0.02 mol% Mo.

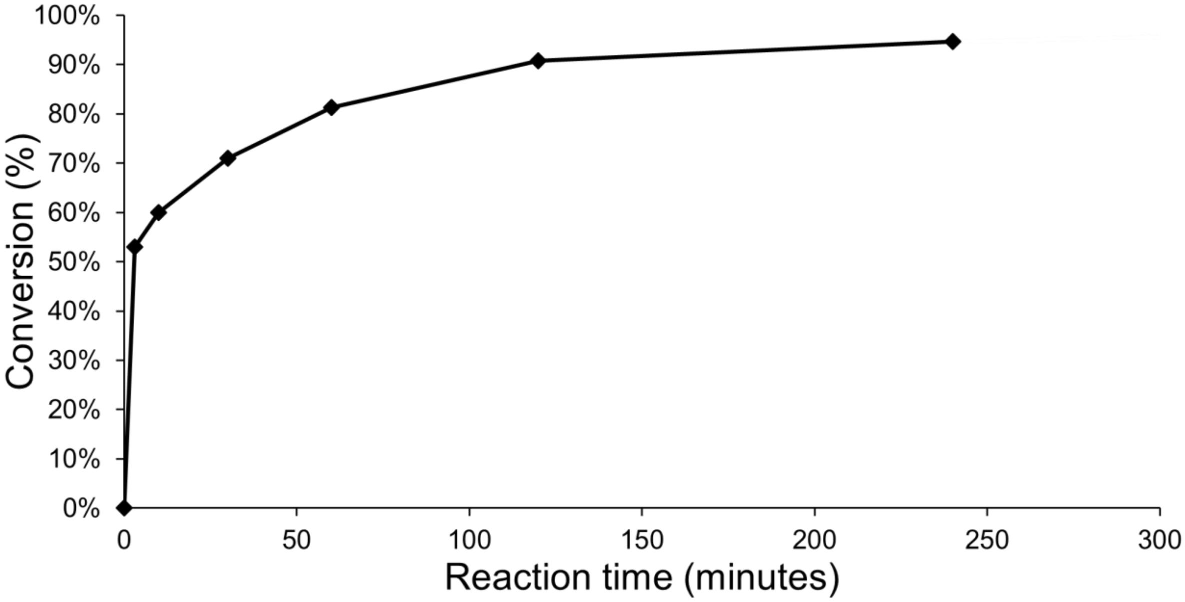

Figure S26. Metathesis of 1-tridecene by **Mo**$^{+}$ at 30 °C. Open reaction vial, ca. 0.02 mol% Mo.

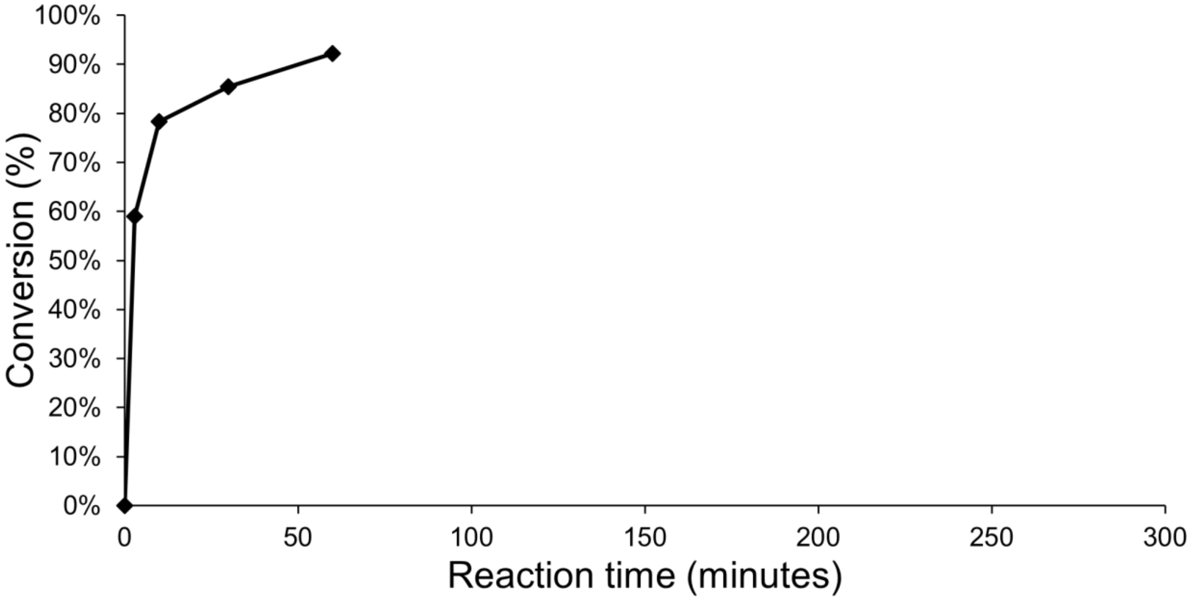

Figure S27. Metathesis of 1-hexadecene by **Mo+** at 30 °C. Open reaction vial, ca. 0.02 mol% Mo.

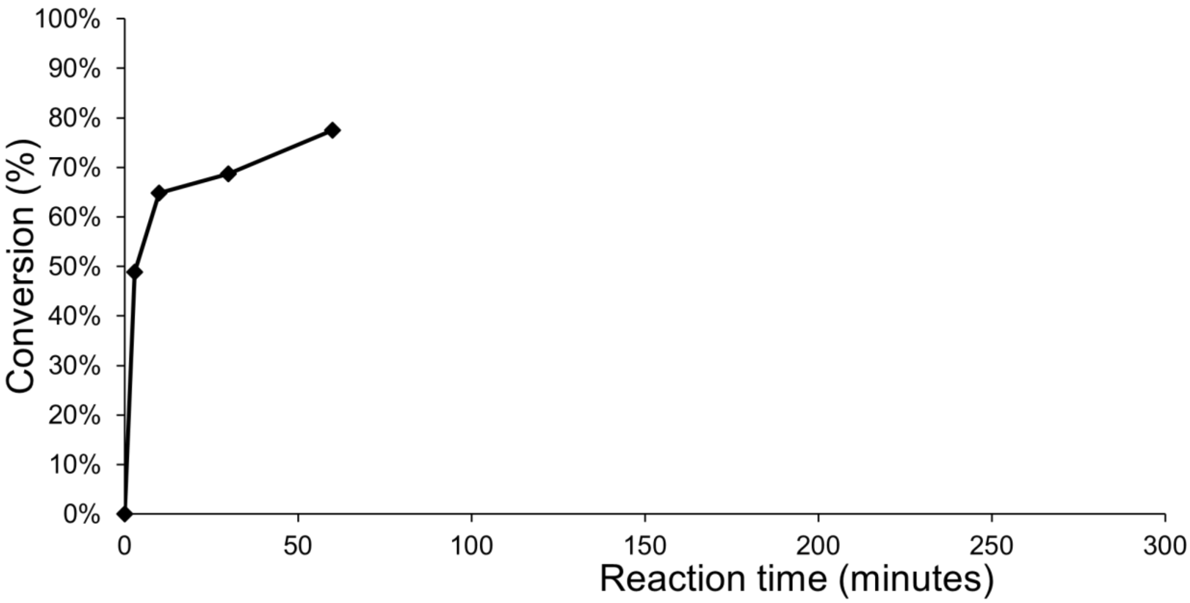

Figure S28. Metathesis of 1-eicosene by **Mo+** at 30 °C. Open reaction vial, ca. 0.04 mol% Mo.

Table S5. Summary of catalytic data for metathesis of linear α-olefins by **Mo**$^{+}$ at 30 °C.[a]

**Substrate**	**Maximum product formation rate (mmol product/[mmol Mo min])**[b]	**Initial product E/Z ratio**[b]	**Substrate conversion after 1 h (%)**	**Selectivity for olefin metathesis product (%)**
1-octene	250	4.31	91	99
1-nonene	350	4.05	96	96
1-tridecane	322	3.8	86	97
1-hexadecane	310	3.81	92	97
1-eicosene	365	-[c]	78	-[c]

[a] Conditions: 30 °C, open reaction vials, 2.5 mL reaction volume, substrate : Mo = 5000 : 1 (ca. 0.02 mol% Mo) for 1-octene, 1-nonene, 1-tridecene, and 1-hexadecene, 2500 substrate : 1 Mo (ca. 0.04 mol% Mo) for 1-eicosene
[b] Measured between 3 and 4.5 minutes reaction time for each catalyst
[c] Product peaks not resolved

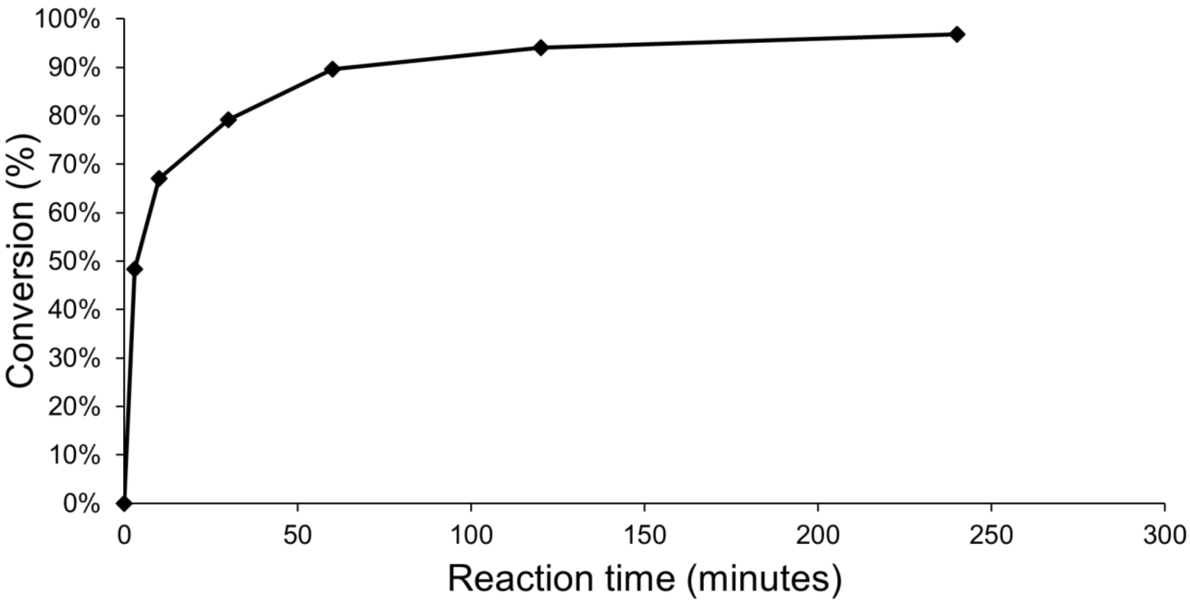

Figure S29. Metathesis of 1-octene by **Mo+/SiO2** at 30 °C. Open reaction vial, ca. 0.02 mol% Mo.

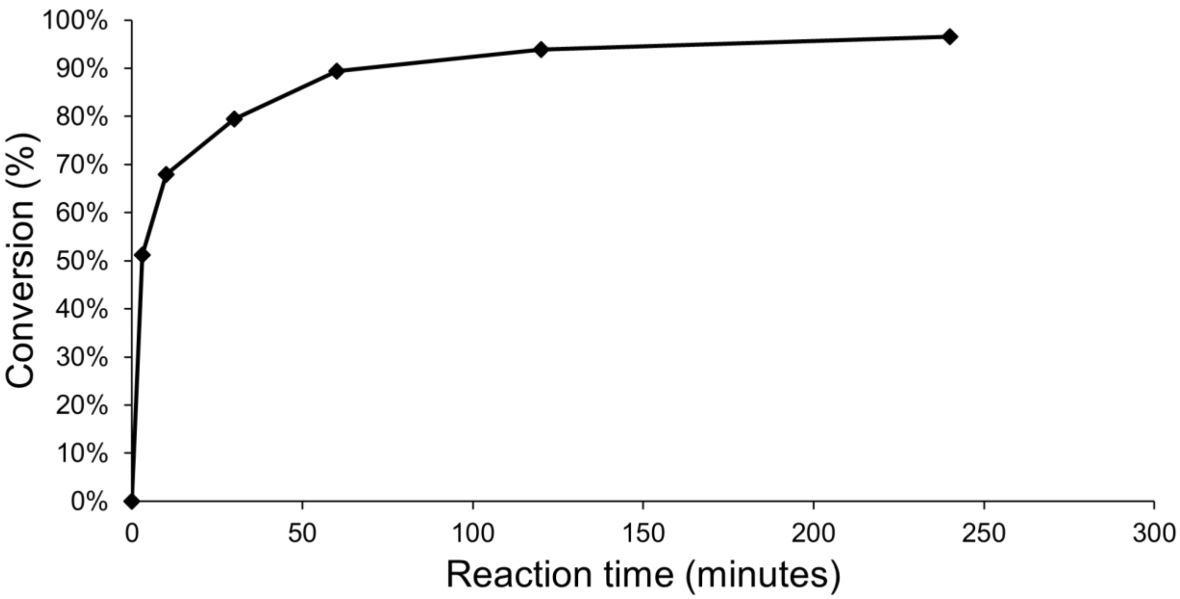

Figure S30. Metathesis of 1-nonene by **Mo+/SiO2** at 30 °C. Open reaction vial, ca. 0.02 mol% Mo.

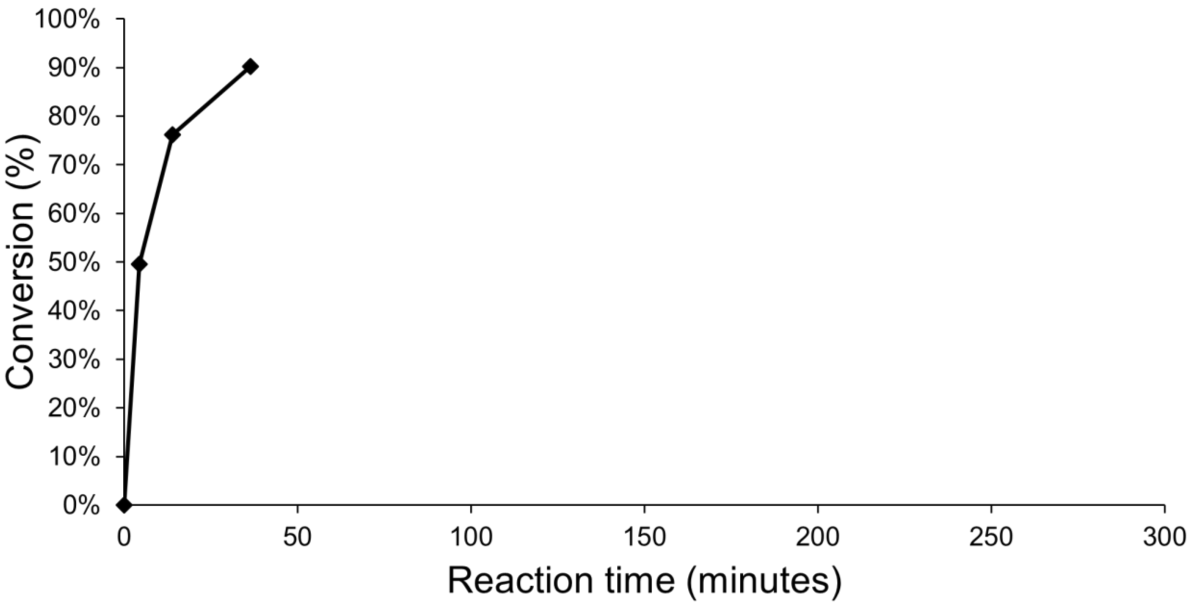

Figure S31. Metathesis of 1-tridecene by **Mo+/SiO2** at 30 °C. Open reaction vial, ca. 0.02 mol% Mo.

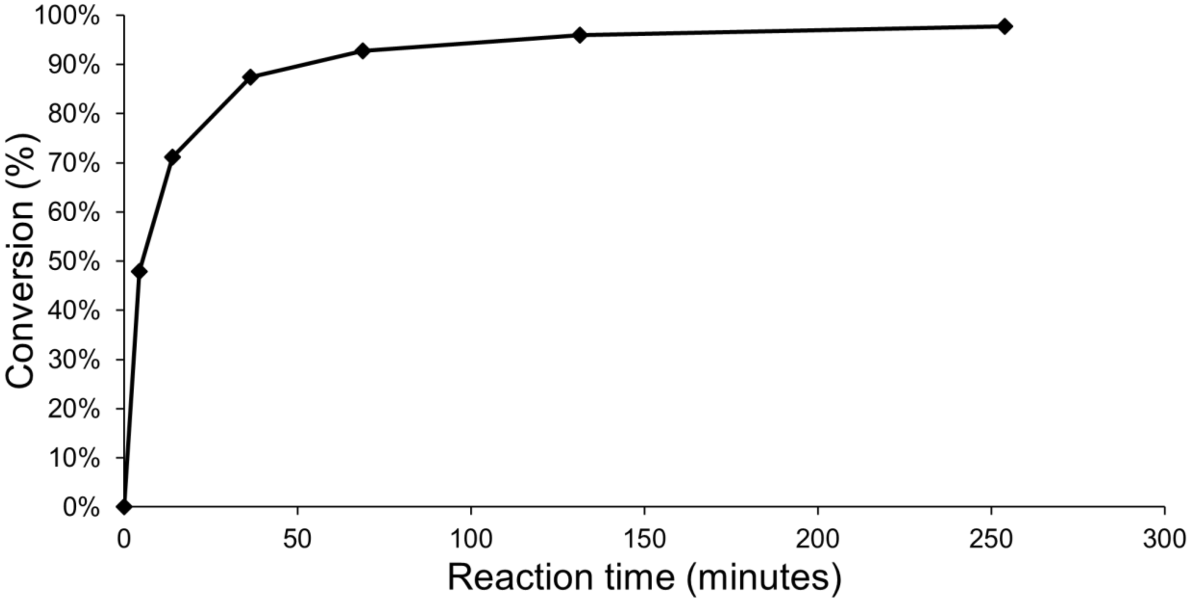

Figure S32. Metathesis of 1-hexadecene by **Mo+/SiO2** at 30 °C. Open reaction vial, ca. 0.02 mol% Mo.

Table S6. Summary of catalytic data for metathesis of linear α-olefins by **Mo^+/SiO_2** at 30 °C.[a]

**Substrate**	**Maximum product formation rate (mmol product/[mmol Mo min])[b]**	**Initial product E/Z ratio[b]**	**Substrate conversion (%) after reaction time in parentheses (minutes)**	**Selectivity for olefin metathesis product (%)**
1-octene	300	4.6	98 (60)	95
1-nonene	460	3.2	95 (60)	96
1-tridecene	380	3.8	90 (30)	97
1-hexadecene	250	3.8	93 (60)	97
1-eicosene	365	-[c]	40 (3)	-[c]

[a] Conditions: 30 °C, open reaction vials, 2.5 mL reaction volume, substrate : Mo = 5000 : 1 (ca. 0.02 mol% Mo) for 1-octene, 1-nonene, 1-tridecene, and 1-hexadecene, 2500 substrate : 1 Mo (ca. 0.04 mol% Mo) for 1-eicosene
[b] Measured between 3 and 4.5 minutes reaction time for each catalyst
[c] Product peaks not resolved

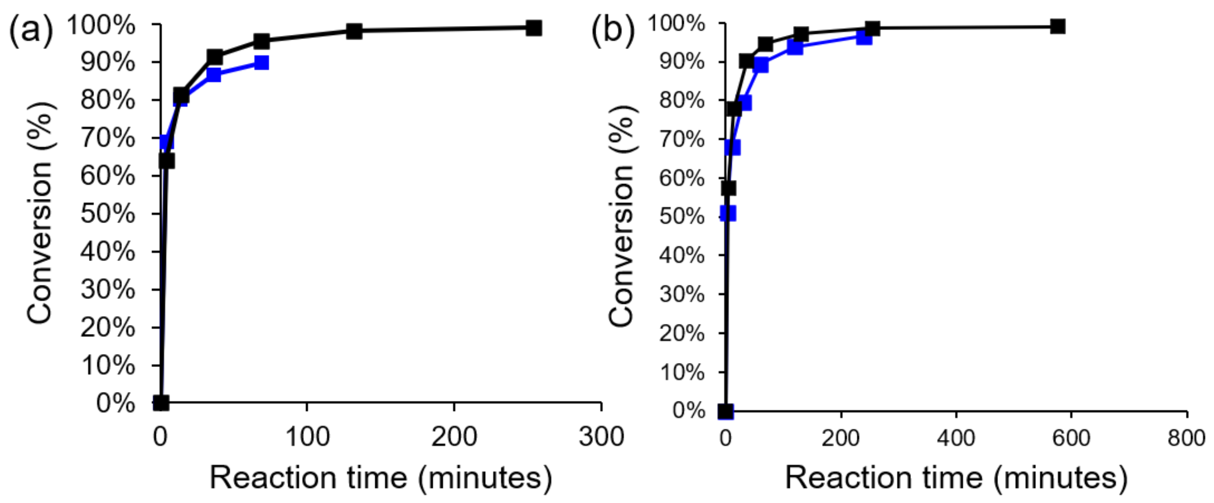

Figure S33. Comparison of two separate 1-nonene metathesis reaction tests by (a) **Mo^+** (b) **Mo^+/SiO_2**. Open reaction vials, ca. 0.02 mol% Mo. TOF_{3min} values from the two tests are 350 and 330 for **Mo^+** and 400 and 450 for **Mo^+/SiO_2**. Combined with experimental uncertainties, this yields an estimated error of +/- 40 on the TOF values for these highly active catalysts.

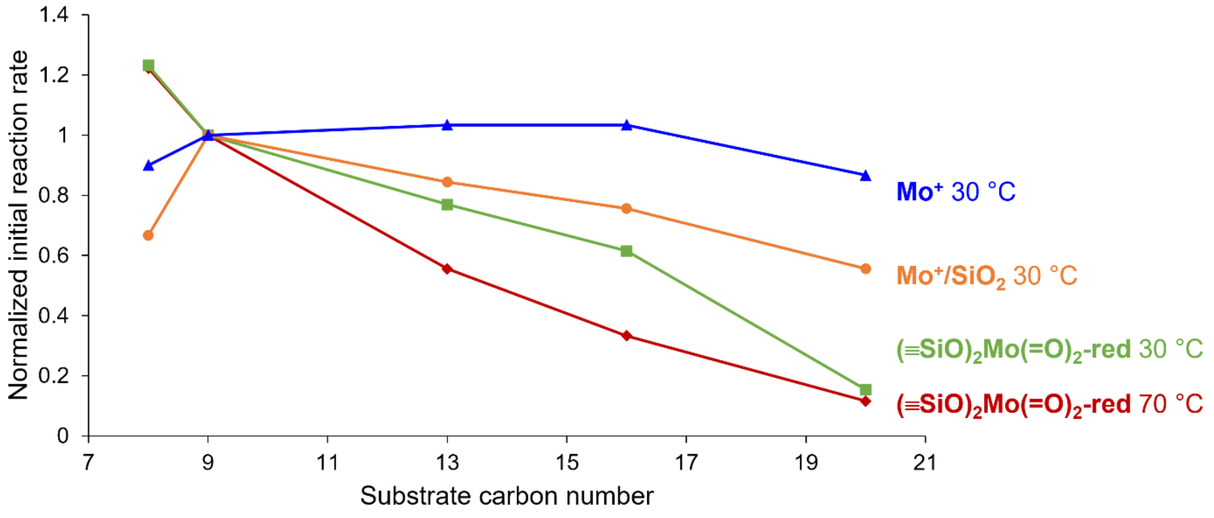

Figure S34. Comparison of reactivity trends for **Mo^+** at 30 °C (blue), **Mo^+/SiO_2** at 30 °C (orange), **$(\equiv SiO)_2Mo(=O)_2$-red** at 30 °C (green), and **$(\equiv SiO)_2Mo(=O)_2$-red** at 70 °C (red) as a function of substrate carbon number. For each catalyst, the initial reaction rate was normalized by that of 1-nonene.

Spectroscopic and elemental analyses of post-reaction catalysts

Table S7. Elemental analyses of **$(\equiv SiO)_2Mo(=O)_2$-red** before and after 1-hexadecene metathesis at 30 °C[a]

**Material**	**Reaction time (h) (% conv.)**	**Carbon content (wt%)**	**Molecules per nm^2**	**Minimum fractional surface coverage[b]**	**Maximum fractional surface coverage[c]**
SiO_{2-700} contacted with 15-triacontene	-	3.3	0.28	0.2	0.5
**$(\equiv SiO)_2Mo(=O)_2$-red**	0 (0)	0.9[d]	-	-	-
**$(\equiv SiO)_2Mo(=O)_2$-red**	1 (8)	1.8	0.15	0.1	0.3
**$(\equiv SiO)_2Mo(=O)_2$-red**	3 (35)	5.0	0.42	0.3	0.7

[a] Conditions: 30 °C, open reaction vials, 3 mL reaction volume, substrate : Mo = 1000 : 1
[b] Estimated assuming the sole organic on the surface is 15-triacontene, with a minimal projected area of 0.75 nm^2 and a surface area of 200 m^2/g
[c] Estimated assuming the sole organic on the surface is 15-triacontene, with a maximal projected area of 1.7 nm^2 and a surface area of 200 m^2/g
[d] Arising from residual -TMS groups of the organosilicon reductant.

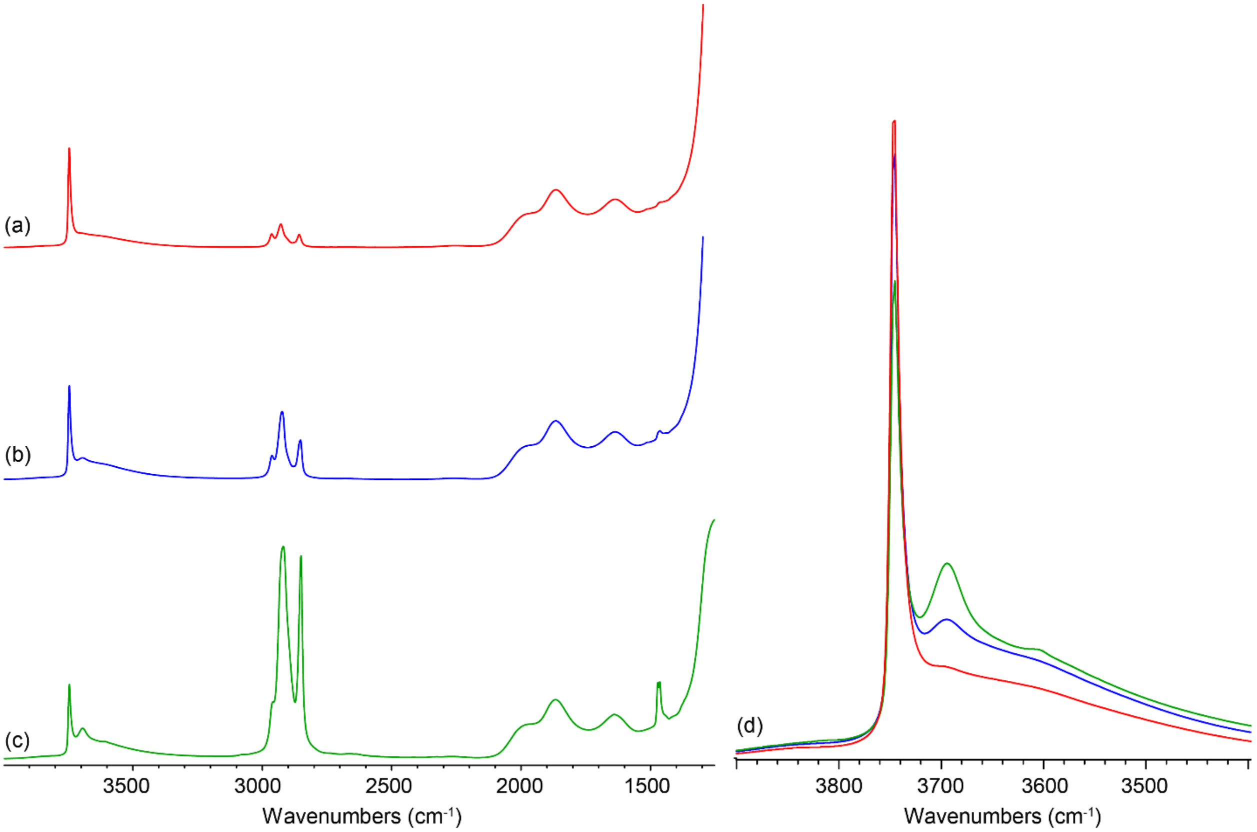

Figure S35. Transmission FTIR spectra of **(≡SiO)$_2$Mo(O)$_2$-red** (a) before and after 1-hexadecene metathesis at 30 °C for (b) 1 hour (ca. 8% conversion) or (c) 3 hours (ca. 35% conversion). Each material was washed 3x with benzene and dried overnight at 10^{-5} mbar. (d) Expansion and overlay of the -OH stretch region for the three spectra in (a-c), showing the decrease in intensity of the signal from isolated SiOH at 3745 cm^{-1} with concomitant emergence of broad signals at 3695 and 3606 cm^{-1}, consistent with interactions of isolated SiOH species with adsorbed olefin metathesis products.

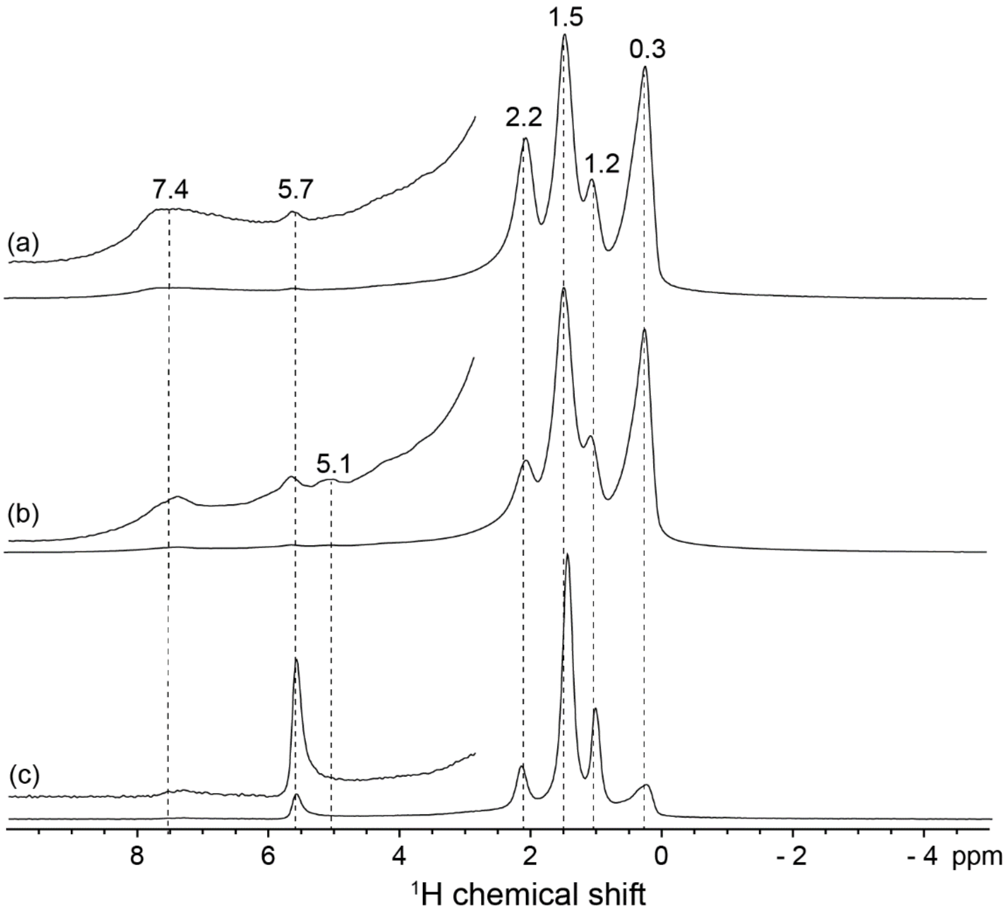

Figure S36. Solid-state 1D ^{1}H echo MAS NMR spectrum of **(≡SiO)$_2$Mo(O)$_2$-red** after 1-hexadecene metathesis at 30 °C for (a) 1 hour (ca. 8% conversion), (b) 3 hours (ca. 35% conversion), or (c) 24 hours (ca. 80% conversion), followed by washing 3x with benzene and drying overnight at 10^{-5} mbar. The spectra were acquired at 40 kHz MAS, 16.4 T, and 298 K.

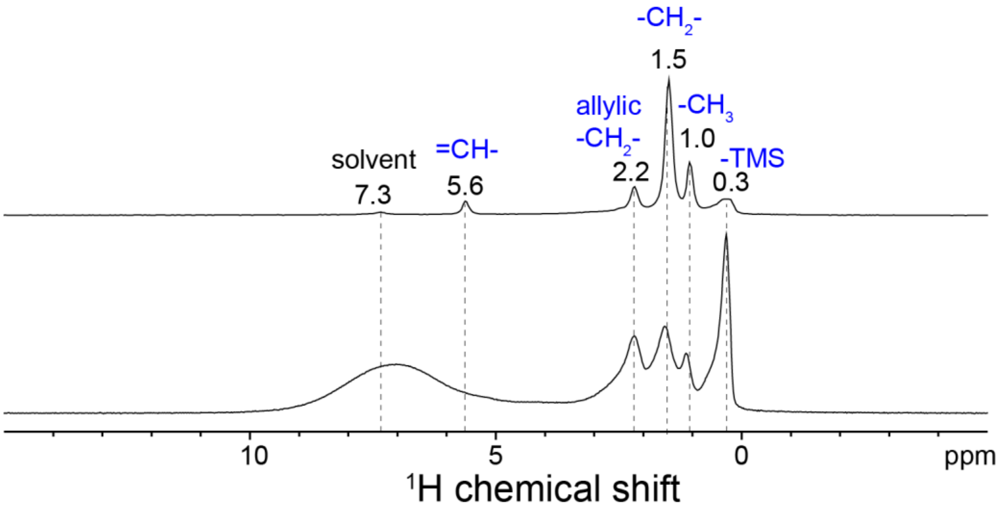

Figure S37. Solid-state ^{1}H MAS NMR echo spectra of **(≡SiO)$_2$Mo(=O)$_2$-red** after 24 h reaction with 1-hexadecene (top) or 1-nonene (bottom) in 1,2-dichlorobenzene at 30 °C, 3x washing with C_6H_6, and drying under high vacuum. The spectra were acquired at 16.4 T, 50 kHz MAS, and 280 K. After reaction with 1-nonene, a much more prominent signal is observed at 7.3 ppm from aromatic moieties of solvent molecules compared to after reaction with 1-hexadecene. This suggests that physisorption of solvent molecules is competitive with that of the olefin metathesis product molecules.

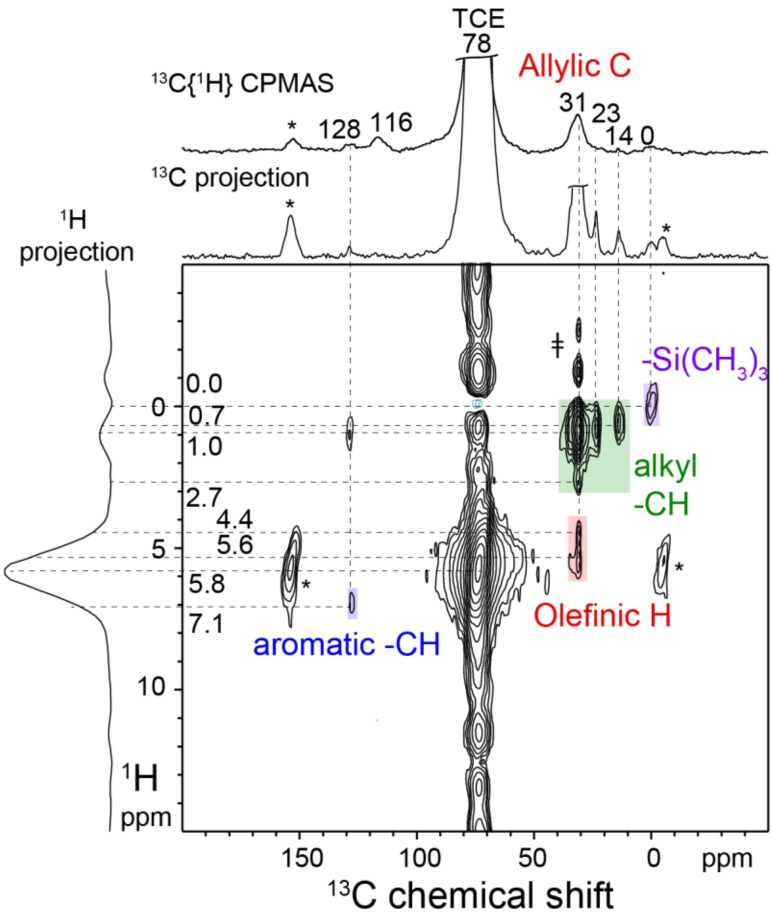

Figure S38. Solid-state 2D $^{13}C\{^1H\}$ DNP-HETCOR spectra of **(≡SiO)$_2$Mo(=O)$_2$-red** after 4 h reaction with 1-hexadecene in 1,2-dichlorobenzene at 30 °C, 3x washing with C_6H_6, and drying under high vacuum. The 2D spectrum was acquired at 14.1 T, 12.5 kHz MAS, 100 K, under continuous microwave irradiation at 395 GHz, in the presence of 16 mM TEKPol biradical in 1,1,2,2-tetrachloroethane (DNP solvent), and with a ^{13}C-^1H contact time of 1 ms. A 1D $^{13}C\{^1H\}$ DNP-CPMAS spectrum acquired under the same conditions is shown along the horizontal axis for comparison with the 1D ^{13}C projection of the 2D spectrum.

Discussion of signal assignments for Figure S38

The ^1H signal at 0.0 ppm is assigned to -TMS moieties based on its correlation in Fig. S36 to a ^{13}C signal at 0 ppm (purple shaded region in Figure S36). The ^1H signals at 0.7 to 2.7 ppm are assigned to aliphatic moieties (green shaded regions in Fig. 5 and S36) based on their correlation to ^{13}C signals at 14, 23, and 31 ppm. The ^1H signals from 5.8 to 6.4 ppm (grey shaded regions) are assigned to DNP solvent molecules (1,1,2,2-tetrachloroethane) based on their correlation in Fig. S36 to ^{13}C signals at 78 ppm. The ^1H signals from 4.4 to 5.6 ppm are correlated in Fig. S36 to the

^{13}C signal at 31 ppm from allylic ^{13}C moieties and are assigned to olefinic moieties. There are no correlations of these signals to the ^{13}C signals from olefinic moieties at 116 and 128 ppm observed in Fig. S36 likely because of their low signal intensity and spectral broadening under the low-temperature measurement conditions. Nevertheless, the signal assignments are corroborated by the correlation of the ^{1}H signal at 5.6 ppm to the ^{13}C signal at 132 ppm in the $^{1}H\{^{13}C\}$ *D*-HMQC spectrum acquired at room temperature in Figure 4. Finally, the ^{1}H signals between 7.1 to 8.5 ppm are assigned to aromatic species (e.g., 1,2-dichlorobenzene, blue shaded regions) based on the weak correlation of the ^{1}H signal at 7.1 ppm to the ^{13}C signal at 128 ppm in Figure S36. We note that ^{1}H signals at >7 ppm could also arise in part from strongly hydrogen-bonded or hydrated -OH moieties, as have been previously observed under DNP-NMR conditions for silicate and aluminosilicate zeolites[49,50] and other silica based materials,[51] though such species are expected to be dilute in the dehydroxylated supported catalyst **$(\equiv SiO)_2Mo(=O)_2$-red**.

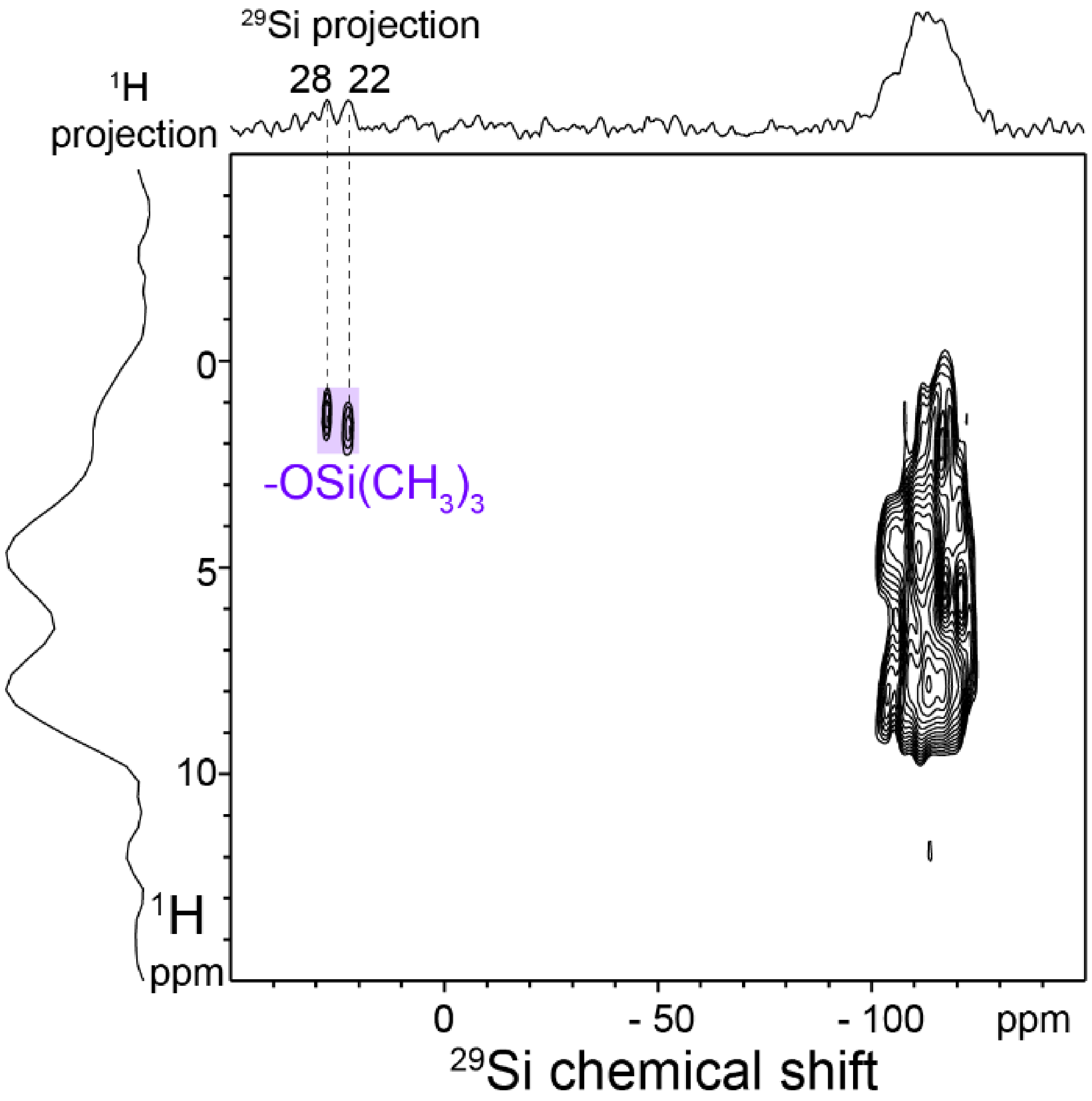

Figure S39. Solid-state 2D ^{29}Si{^{1}H} DNP-HETCOR spectrum of **(≡SiO)$_2$Mo(=O)$_2$-red** after 4 h reaction with 1-hexadecene in 1,2-dichlorobenzene at 30 °C, 3x washing with C_6H_6, and drying under high vacuum. The 2D spectrum was acquired at 14.1 T, 12.5 kHz MAS, 100 K, under continuous microwave irradiation at 395 GHz, in the presence of 16 mM TEKPol biradical in 1,1,2,2-tetrachloroethane (DNP solvent), and with a ^{29}Si-^{1}H contact time of 5 ms. (Same spectrum as Figure 5b in the main text with an expanded frequency axis showing the signals arising from surface -OTMS moieties).

Table S8. ^{1}H T_2 relaxation analyses extracted from solid-state ^{1}H CPMG spectra of **(≡SiO)$_2$Mo(=O)$_2$-red** after 24 h reaction with 1-hexadecene or 1-nonene in 1,2-dichlorobenzene at 30 °C, 3x washing with C_6H_6, and drying under high vacuum. The spectra were acquired at 16.4 T, 50 kHz MAS, 280 K, and with varying numbers of CPMG loops. The integrated signal intensities were extracted using the DMFIT software[30] and fit to stretched exponential fitting functions[31] to extract the characteristic T_2 relaxation rate of the corresponding ^{1}H species and the stretched exponent, β.

**^{1}H signal position**	**Assignment**	**T_2 (ms)**	β
**(≡SiO)$_2$Mo(=O)$_2$-red** after 1-hexadecene metathesis			
0.3	-SiCH_3	7.2	0.35
1.0	-CH_3	2.6	0.88
1.5	Aliphatic -CH_2-	2.3	0.90
2.2	Allylic -CH_2-	1.9	0.77
5.6	Olefinic -CH=	1.9	0.80
7.3	Physisorbed solvent	0.1	0.27
**(≡SiO)$_2$Mo(=O)$_2$-red** after 1-nonene metathesis			
0.3	-SiCH_3	26.9	0.62
1.0	-CH_3	2.3	0.48
1.5	Aliphatic -CH_2-	1.3	0.45
2.2	Allylic -CH_2-	1.5	0.36
7.3	Physisorbed solvent	0.3	0.14

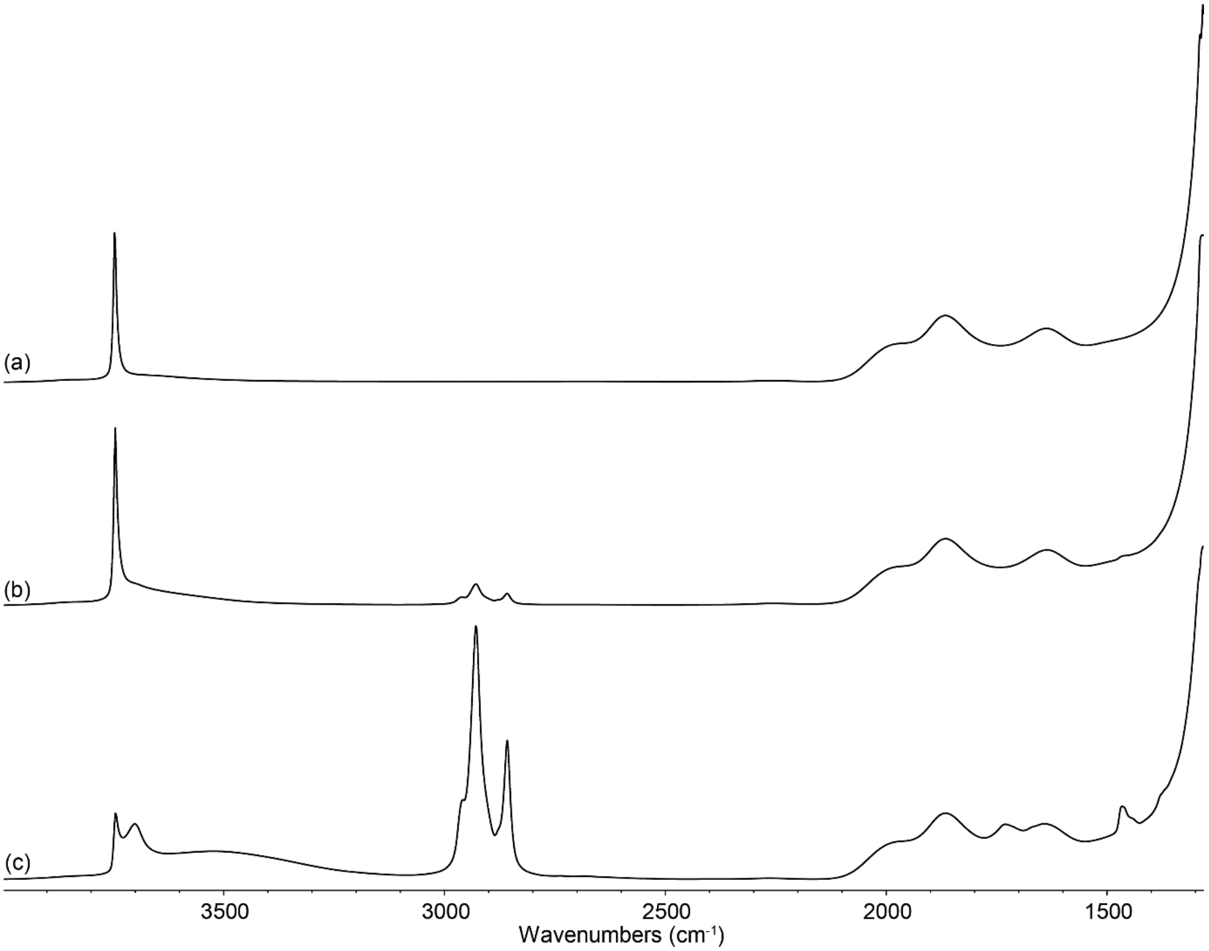

Figure S40. Transmission FTIR spectra of SiO_{2-700} (a) as prepared or contacted for 3 hours with (b) 1 M 1-hexadecene or (c) 0.3 M 15-triacontene (distilled product of 1-hexadecene metathesis) in dichlorobenzene followed by washing 3x with benzene and drying overnight at 10^{-5} mbar.

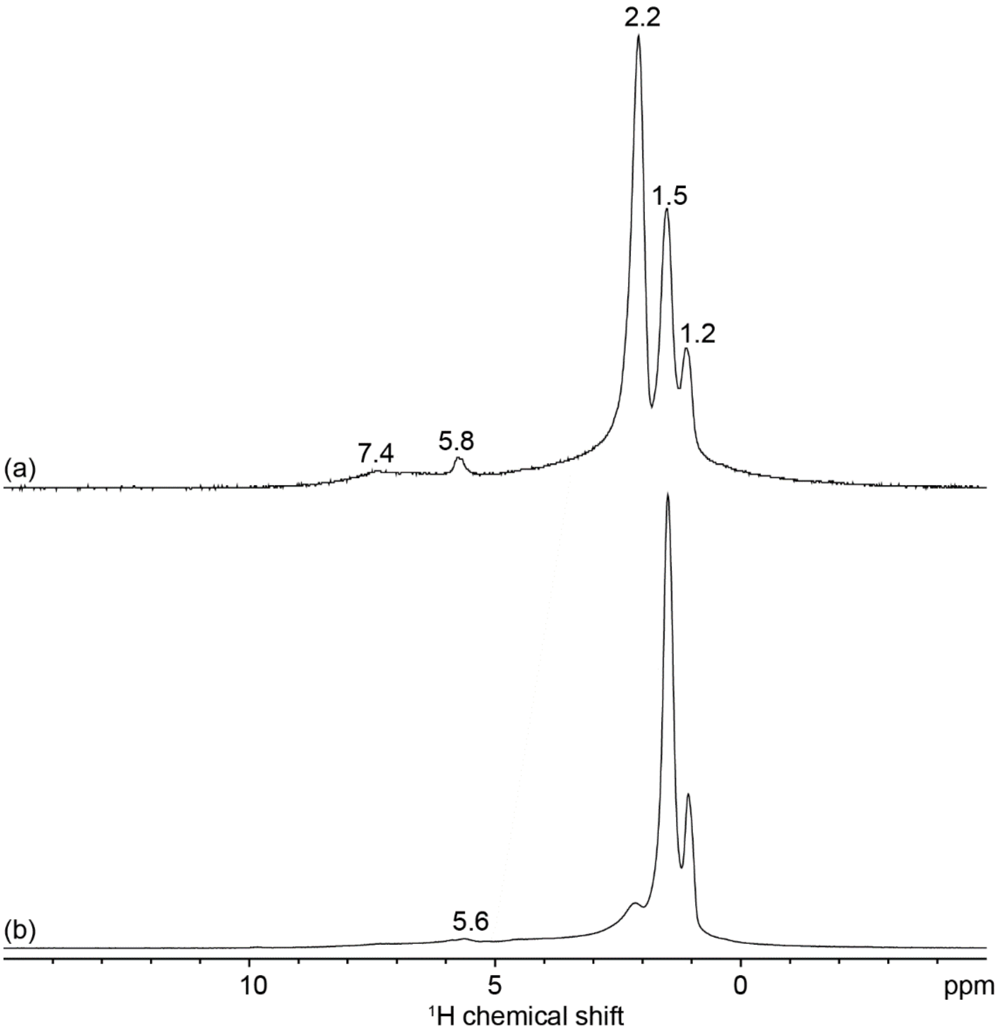

Figure S41. Solid-state 1D ^{1}H echo MAS NMR spectrum of SiO_{2-700} contacted for 3 hours with (a) 1 M 1-hexadecene or (b) 0.3 M 15-triacontene (distilled product of 1-hexadecene metathesis) in dichlorobenzene followed by washing 3x with benzene and drying overnight at 10^{-5} mbar.

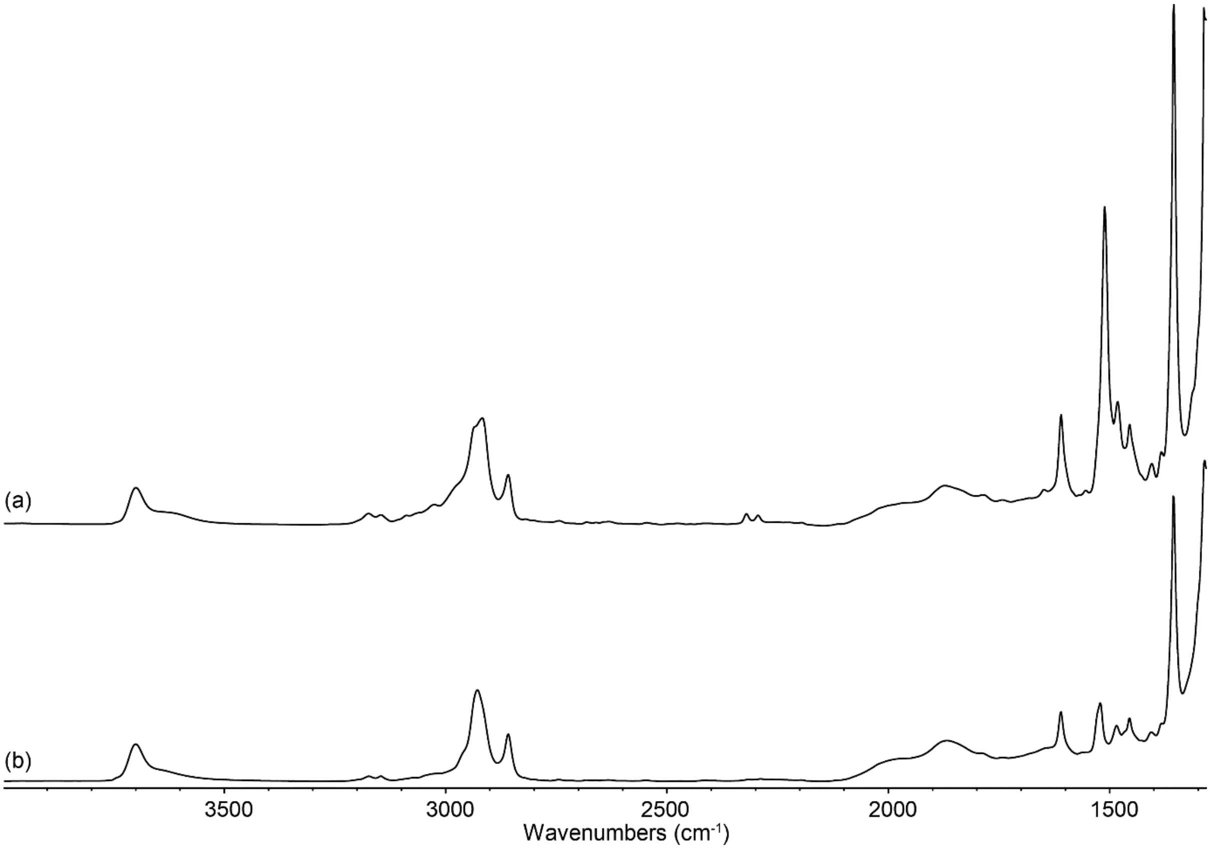

Figure S42. Transmission FTIR spectra of **Mo⁺/SiO₂** (a) before and (b) after 1-hexadecene metathesis at 30 °C for 1 hour, followed by washing 3x with benzene and drying overnight at 10^{-5} mbar. Signals at 2320 and 2295 cm^{-1} from acetonitrile are removed, indicating decoordination of acetonitrile under reaction conditions. Signals in the 1300-1600 cm^{-1} region are also reduced in intensity likely due to remove of the aromatic –$C(Me)_2Ph$ alkylidene moiety on catalyst initiation. Notably, the signals from SiOH species at 3700 and 3615 cm^{-1} are minimally perturbed after reaction, indicating that these OH sites participate little in olefin metathesis product adsorption.

Summary of MD simulation results

Table S9. Summary of AIMD simulation results of olefin interactions with a model for amorphous silica.

Compound	Average adsorption energy[a] (kJ/mol)	Lowest adsorption energy[a] (kJ/mol)	Distances between vinylic H atoms of olefin and closest O atom of surface OH (Å)[b]		Distances between vinylic H atoms of olefin and O atoms of surface siloxane bridges (Å)[b]		
			1	2	1	2	3
1-propene	-114.91	-123.47	2.70	4.82	2.84	3.24	3.50
1-hexene	-144.85	-167.38	3.37	3.44	2.82	2.91	2.92
1-octene	-132.81	-138.81	3.78	4.18	2.84	4.07	4.10
1-nonene	-127.80	-139.44	4.21	4.63	3.13	3.47	3.61
1-decene	-157.57	-175.65	2.35	2.46	3.27	3.30	3.61
E-2-butene	-129.40	-136.64	2.38	2.44	2.91	3.02	3.30
E-3-hexene	-138.21	-147.68	2.36	2.39	3.35	3.35	3.57
E-4-octene	-123.27	-140.15	4.30	4.32	2.98	3.17	3.28
E-5-decene	-148.74	-157.99	2.84	3.25	3.39	3.41	3.74
Z-5-decene	-153.60	-173.16	2.74	2.91	2.43	3.44	3.61

[a] Of the four lowest energy structures found for each olefin

[b] From lowest energy structure found for each olefin

References

[1] T. Saito, H. Nishiyama, H. Tanahashi, K. Kawakita, H. Tsurugi, K. Mashima, '1,4-Bis(trimethylsilyl)-1,4-diaza-2,5-cyclohexadienes as strong salt-free reductants for generating low-valent early transition metals with electron-donating ligands', *J. Am. Chem. Soc.* **2014**, *136*, 5161–5170.

[2] K. Yamamoto, K. W. Chan, V. Mougel, H. Nagae, H. Tsurugi, O. V. Safonova, K. Mashima, C. Copéret, 'Silica-supported isolated molybdenum di-oxo species: Formation and activation with organosilicon agent for olefin metathesis', *Chem. Commun.* **2018**, *54*, 3989–3992.

[3] J. Jarupatrakorn, M. P. Coles, T. D. Tilley, 'Synthesis and characterization of MO[OSi(OtBu)3] 4 and MO2[OSt(O≤Bu)3]2 (M = Mo, W): Models for isolated oxo-molybdenum and -tungsten sites on silica and precursors to molybdena- And tungsta-silica materials', *Chem. Mater.* **2005**, *17*, 1818–1828.

[4] E. L. Lee, I. E. Wachs, 'In situ spectroscopic investigation of the molecular and electronic structures of SiO 2 supported surface metal oxides', *J. Phys. Chem. C* **2007**, *111*, 14410–14425.

[5] M. R. Buchmeiser, S. Sen, C. Lienert, L. Widmann, R. Schowner, K. Herz, P. Hauser, W. Frey, D. Wang, 'Molybdenum Imido Alkylidene N-Heterocyclic Carbene Complexes: Structure–Productivity Correlations and Mechanistic Insights', *ChemCatChem* **2016**, *8*, 2710–2723.

[6] M. Pucino, M. Inoue, C. P. Gordon, R. Schowner, L. Stöhr, S. Sen, C. Hegedüs, E. Robé, F. Tóth, M. R. Buchmeiser, C. Copéret, 'Promoting Terminal Olefin Metathesis with a Supported Cationic Molybdenum Imido Alkylidene N-Heterocyclic Carbene Catalyst', *Angew. Chemie - Int. Ed.* **2018**, *57*, 14566–14569.

[7] P. A. Zhizhko, V. Mougel, J. De Jesus Silva, C. Copéret, 'Benchmarked Intrinsic Olefin Metathesis Activity: Mo vs. W', *Helv. Chim. Acta* **2018**, *101*, e1700302.

[8] A. Brinkmann, A. P. M. Kentgens, 'Proton-selective O-17-H-1 distance measurements in fast magic-angle-spinning solid-state NMR spectroscopy for the determination of hydrogen bond lengths', *J. Am. Chem. Soc.* **2006**, *128*, 14758–14759.

[9] S. Meiboom, D. Gill, 'Modified spin-echo method for measuring nuclear relaxation times', *Rev. Sci. Instrum.* **1958**, *29*, 688–691.

[10] A. Zagdoun, G. Casano, O. Ouari, M. Schwarzwälder, A. J. Rossini, F. Aussenac, M. Yulikov, G. Jeschke, C. Copéret, A. Lesage, P. Tordo, L. Emsley, 'Large Molecular Weight Nitroxide Biradicals Providing Efficient Dynamic Nuclear Polarization at Temperatures up to 200 K', *J. Am. Chem. Soc.* **2013**, *135*, 12790–12797.

[11] B. Elena, G. de Paëpe, L. Emsley, 'Direct spectral optimisation of proton-proton homonuclear dipolar decoupling in solid-state NMR', *Chem. Phys. Lett.* **2004**, *398*, 532–538.

[12] B. M. Fung, A. K. Khitrin, K. Ermolaev, 'An Improved Broadband Decoupling Sequence for Liquid Crystals and Solids', *J. Magn. Reson.* **2000**, *142*, 97–101.

[13] A. D. Becke, 'A new mixing of Hartree-Fock and local density-functional theories', *J. Chem. Phys.* **1993**, *98*, 1372–1377.

[14] A. D. Becke, 'Density-functional thermochemistry. III. The role of exact exchange', *J. Chem. Phys.* **1993**, *98*, 5648–5652.

[15] C. Lee, W. Yang, R. G. Parr, 'Development of the Colle-Salvetti correlation-energy formula into a functional of the electron density', *Phys. Rev. B* **1988**, *37*, 785–789.

[16] S. H. Vosko, L. Wilk, M. Nusair, 'Accurate spin-dependent electron liquid correlation energies for local spin density calculations: a critical analysis', *Can. J. Phys.* **1980**, *58*, 1200–1211.

[17] P. J. Stephen, F. J. Devlin, C. F. Chabalowski, M. J. Frisch, 'Ab Initio Calculation of Vibrational Absorption', *J. Phys. Chem.* **1994**, *98*, 11623–11627.

[18] W. J. Hehre, R. Ditchfield, J. A. Pople, 'Self—Consistent Molecular Orbital Methods. XII. Further Extensions of Gaussian—Type Basis Sets for Use in Molecular Orbital Studies of Organic Molecules', *J. Chem. Phys.* **1972**, *56*, 2257–2261.

[19] M. J. Frisch, G. W. Trucks, H. B. Schlegel, G. E. Scuseria, M. A. Robb, J. R. Cheeseman, G. Scalmani, V. Barone, G. A. Petersson, H. Nakatsuji, X. Li, M. Caricato, A. Marenich, J. Bloino, B. G. Janesko, R. Gomperts, B. Mennucci, H. P. Hratchian, J. V. Ortiz, A. F. Izmaylov, J. L. Sonnenberg, D. Williams-Young, F. Ding, F. Lipparini, F. Egidi, J. Goings, B. Peng, A. Petrone,

T. Henderson, D. Ranasinghe, V. G. Zakrzewski, J. Gao, N. Rega, G. Zheng, W. Liang, M. Hada, M. Ehara, K. Toyota, R. Fukuda, J. Hasegawa, M. Ishida, T. Nakajima, Y. Honda, O. Kitao, H. Nakai, T. Vreven, K. Throssell, J. A. Montgomery, J. E. Peralta, F. Ogliaro, M. Bearpark, J. J. Heyd, E. Brothers, K. N. Kudin, V. N. Staroverov, T. Keith, R. Kobayashi, J. Normand, K. Raghavachari, A. Rendell, J. C. Burant, S. S. Iyengar, J. Tomasi, M. Cossi, J. M. Millam, M. Klene, C. Adamo, R. Cammi, J. W. Ochterski, R. L. Martin, K. Morokuma, O. Farkas, J. B. Foresman, D. J. Fox, 'Gaussian Revision D.01' 2016.

[20] T. D. Kühne, M. Iannuzzi, M. Del Ben, V. V. Rybkin, P. Seewald, F. Stein, T. Laino, R. Z. Khaliullin, O. Schütt, F. Schiffmann, D. Golze, J. Wilhelm, S. Chulkov, M. H. Bani-Hashemian, V. Weber, U. Borštnik, M. Taillefumier, A. S. Jakobovits, A. Lazzaro, H. Pabst, T. Müller, R. Schade, M. Guidon, S. Andermatt, N. Holmberg, G. K. Schenter, A. Hehn, A. Bussy, F. Belleflamme, G. Tabacchi, A. Glöß, M. Lass, I. Bethune, C. J. Mundy, C. Plessl, M. Watkins, J. VandeVondele, M. Krack, J. Hutter, 'CP2K: An electronic structure and molecular dynamics software package -Quickstep: Efficient and accurate electronic structure calculations', *J. Chem. Phys.* **2020**, *152*, 194103.

[21] J. P. Perdew, K. Burke, M. Ernzerhof, 'Generalized Gradient Approximation Made Simple', *Phys. Rev. Lett.* **1996**, *77*, 3865–3868.

[22] J. P. Perdew, A. Ruzsinszky, G. I. Csonka, O. A. Vydrov, G. E. Scuseria, L. A. Constantin, X. Zhou, K. Burke, 'Erratum: Restoring the density-gradient expansion for exchange in solids and surfaces (Physical Review Letters (2008) 100 (136406))', *Phys. Rev. Lett.* **2009**, *102*, 39902.

[23] Y. Zhang, W. Yang, 'Comment on "generalized gradient approximation made simple"', *Phys. Rev. Lett.* **1998**, *80*, 890.

[24] S. Grimme, J. Antony, S. Ehrlich, H. Krieg, 'A consistent and accurate ab initio parametrization of density functional dispersion correction (DFT-D) for the 94 elements H-Pu', *J. Chem. Phys.* **2010**, *132*, 154104.

[25] E. R. Johnson, A. D. Becke, 'A post-Hartree-Fock model of intermolecular interactions: Inclusion of higher-order corrections', *J. Chem. Phys.* **2006**, *124*, 174104.

[26] J. VandeVondele, J. Hutter, 'Gaussian basis sets for accurate calculations on molecular systems in gas and condensed phases', *J. Chem. Phys.* **2007**, *127*, 114105.

[27] S. Goedecker, M. Teter, 'Separable dual-space Gaussian pseudopotentials', *Phys. Rev. B - Condens. Matter Mater. Phys.* **1996**, *54*, 1703–1710.

[28] A. Comas-Vives, 'Amorphous SiO2 surface models: Energetics of the dehydroxylation process, strain, ab initio atomistic thermodynamics and IR spectroscopic signatures', *Phys. Chem. Chem. Phys.* **2016**, *18*, 7475–7482.

[29] G. Bussi, D. Donadio, M. Parrinello, 'Canonical sampling through velocity rescaling', *J. Chem. Phys.* **2007**, *126*, 014101.

[30] D. Massiot, F. Fayon, M. Capron, I. King, S. Le Calvé, B. Alonso, J. O. Durand, B. Bujoli, Z. Gan, G. Hoatson, 'Modelling one- and two-dimensional solid-state NMR spectra', *Magn. Reson. Chem.* **2002**, *40*, 70–76.

[31] D. C. Johnston, 'Stretched exponential relaxation arising from a continuous sum of exponential decays', *Phys. Rev. B - Condens. Matter Mater. Phys.* **2006**, *74*, 184430.
